# NBIA: a network-based integrative analysis framework – applied to pathway analysis

**DOI:** 10.1038/s41598-020-60981-9

**Published:** 2020-03-06

**Authors:** Tin Nguyen, Adib Shafi, Tuan-Minh Nguyen, A. Grant Schissler, Sorin Draghici

**Affiliations:** 1 Department of Computer Science and Engineering, University of Nevada, Reno, 89557 Nevada United States; 2 Department of Mathematics and Statistics, University of Nevada, Reno, 89557 Nevada United States; 3 Wayne State University, Department of Computer Science, Detroit, 48202 Michigan United States

**Keywords:** Microarrays, Statistical methods

## Abstract

With the explosion of high-throughput data, effective integrative analyses are needed to decipher the knowledge accumulated in biological databases. Existing meta-analysis approaches in systems biology often focus on hypothesis testing and neglect real expression changes, i.e. effect sizes, across independent studies. In addition, most integrative tools completely ignore the topological order of gene regulatory networks that hold key characteristics in understanding biological processes. Here we introduce a novel meta-analysis framework, Network-Based Integrative Analysis (NBIA), that transforms the challenging meta-analysis problem into a set of standard pathway analysis problems that have been solved efficiently. NBIA utilizes techniques from classical and modern meta-analysis, as well as a network-based analysis, in order to identify patterns of genes and networks that are consistently impacted across multiple studies. We assess the performance of NBIA by comparing it with nine meta-analysis approaches: Impact Analysis, GSA, and GSEA combined with classical meta-analysis methods (Fisher’s and the additive method), plus the three MetaPath approaches that employ multiple datasets. The 10 approaches have been tested on 1,737 samples from 27 expression datasets related to Alzheimer’s disease, acute myeloid leukemia (AML), and influenza. For all of the three diseases, NBIA consistently identifies biological pathways relevant to the underlying diseases while the other 9 methods fail to capture the key phenomena. The identified AML signature is also validated on a completely independent cohort of 167 AML patients. In this independent cohort, the proposed signature identifies two groups of patients that have significantly different survival profiles (Cox p-value 2 × 10^−6^). The NBIA framework will be included in the next release of BLMA Bioconductor package (http://bioconductor.org/packages/release/bioc/html/BLMA.html).

## Introduction

Microarray and sequencing technologies have transformed biological and medical research by allowing us to monitor the biological systems at the molecular level. Enormous volumes of molecular data have accumulated in public repositories, including Gene Expression Omnibus (GEO)^1^, cBioPortal^2^, and TCGA (http://cancergenome.nih.gov). Regardless of the high-throughput platforms being used, a standard comparative analysis of expression data usually produces a set of differentially expressed (DE) genes, which are often regarded as potential biological markers. These genes are important in classifying and subtyping patients, as well as in identifying entities that may involve in biological processes of the underlying diseases^3–6^. However, taken alone, gene biomarkers are insufficient to reveal biological mechanisms. In order to translate the differential expression to biological knowledge, researchers have been developing knowledge bases^7,8^ that map genes and gene products to known functional modules and regulatory networks. Concurrently, computational approaches have been developed for the identification of biomarkers at the systems level from differential expression^9–14^.

Remarkably, reproducibility poses big challenges in biomarker identification. Due to measurement errors and inherent study bias, analyses of independent datasets studying the same condition often result in distinctively different sets of DE genes^15,16^ and pathways^17^. Therefore, effective data integration is needed to integrate such similar studies to obtain reliable and consistent findings. For this purpose, meta-analyses have been performed at both gene^18–21^ and systems levels^22–24^. These approaches typically analyze individual studies independently to assess the significance of differential expression, either at gene or pathway level. The results from individual studies are then combined using p-value-based meta-analysis methods such as Fisher’s^25^, Stouffer’s^26^, maxP^27^, minP^28^, and addCLT^29^. One of the critical pitfalls of these p-value-based meta-analysis methods is that they neglect the actual expression changes, i.e. effect sizes. This might result in information loss. Although p-value is influenced by effect size, it is also greatly affected by sample size^30^. For datasets with large sample size, a test for differential expression will almost always result in a significant p-value, unless the effect size is exactly zero, which is very unlikely in reality. Simply combining the p-values would likely produce varying degree of false discoveries. In addition, most integrative approaches do not take into consideration the topological order of genes that hold key characteristics in understanding biological processes.

Here we propose Network-Based Integrative Analysis (NBIA), a network-based approach that utilizes techniques from both p-values-based and effect-sizes-based methods to reliably identify genes and pathways that are likely to be impacted by the underlying disease. The meta-analysis of effect sizes accurately estimates the central tendency of expression change for individual genes. The estimated genome-scale expression change allows for topology-aware analysis, in which gene interaction and signal propagation are taken into consideration. This approach transforms the meta-analysis problem into a standard topology-aware pathway analysis problem that has been solved efficiently. We illustrate the performance of NBIA using 1,737 samples from 27 studies related to Alzheimer’s disease, influenza, and acute myeloid leukemia (AML). We compared NBIA with 9 other approaches: Impact Analysis (IA), GSEA, and GSA combined with Fisher’s^25^ and the addCLT method^29^, plus 3 MetaPath approaches^23^. NBIA outperforms existing approaches in identifying biological processes relevant to the disease.

## Methods

**Figure 1:**
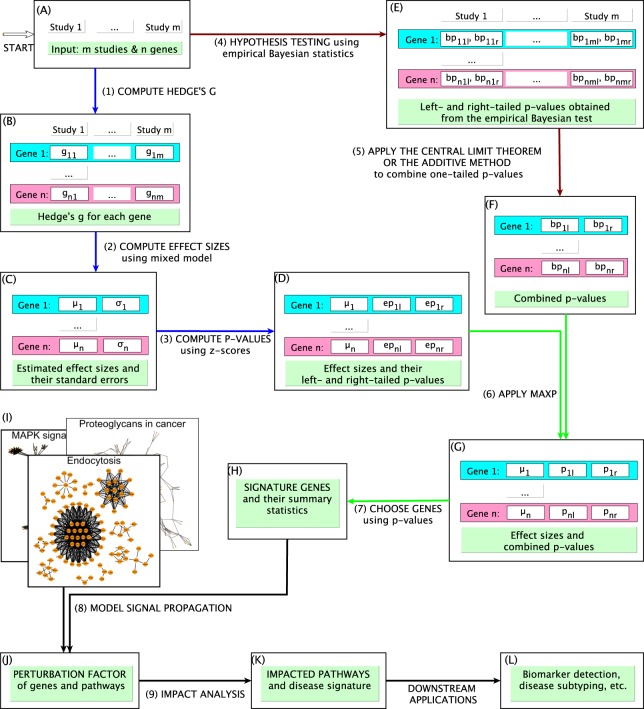
The overall pipeline of NBIA. The input consists of *m* independent datasets and *n* genes. Step (1): calculate effect size (Hedge’s g) for each gene in each study. Step (2): combine effect sizes for each gene across multiple studies using the REstricted Maximum Likelihood (REML) algorithm. Step (3): compute the z-score (${z}_{i}=\frac{{\mu }_{i}}{{\sigma }_{i}}$) and calculate the left- and right-tailed p-values (*ep*_*il*_ and *ep*_*ir*_) using the standard normal distribution. This ends the first module. Step (4): perform hypothesis testing at gene level using empirical Bayesian statistics. For gene *i*^*th*^ and dataset *j*^*th*^, the left- and right-tailed p-values obtained from the Bayesian test are *bp*_*ijl*_ and *bp*_*ijr*_. Step (5): combine the one-tailed p-values for each gene, i.e., *bp*_*il*_ = *addCLT*(*bp*_*i*1*l*_, …, *bp*_*iml*_) and *bp*_*ir*_ = *addCLT*(*bp*_*i*1*r*_, …, *bp*_*imr*_). This ends the second module. Step (6): combine Bayesian p-values with the p-values of the effect size using maxP, i.e. *p*_*il*_ = *max*(*ep*_*il*_, *bp*_*il*_) and *p*_*ir*_ = *max*(*ep*_*ir*_, *bp*_*ir*_). Step (7): choose genes that are significantly impacted from both hypothesis testing and effect size perspectives using FDR-adjusted p-values (1% threshold by default). This ends the third module. Step (8): compute the perturbation factors for NBIA-prioritized genes and pathways. Step (9): identify impacted pathways using impact analysis.

The overall pipeline consists of four main modules: (i) estimating the expression changes (i.e. standardized mean difference), standard errors, and their p-values, (ii) computing the p-values obtained from standard hypothesis testing, (iii) combining the two types of evidence to identify impacted genes and their summary statistics, and finally (iv) performing a network-based pathway analysis. The output is a set of impacted pathways and gene patterns that are consistently impacted across independent studies. These can serve as the disease signature for other downstream analyses. In Fig. 1, the brown arrows show the steps of the first module while the blue and green arrows display the steps of the second and third modules, respectively. The black arrows show the steps of the fourth module, which integrates the computed statistics and the pathway knowledge to identify the biological processes that are impacted or disrupted by the disease.

To estimate the effect sizes of genes across all studies, we first compute standardized mean difference (SMD) for each gene in individual studies. We next estimate the overall effect size and standard error using the random-effects model^31^. This overall effect size represents the gene’s expression change under the effect of the condition. We then calculate the z-scores and the p-values of observing such effect sizes. Concurrently, we also calculate the p-values obtained from classical hypothesis testing. By default, we apply the linear model and empirical Bayesian testing provided by limma^32^ to compute the p-values for differential expression. The two-tailed p-values are converted to one-tailed p-values (left- and right-tailed). For each gene, the one-tailed p-values across all datasets are then combined using the addCLT method^29^. These p-values represent how likely the differential expression is observed by chance.

In the third module, we combine the two types of evidence (one p-value from empirical Bayesian statistics, and one p-value from effect size and standard error). We want that if a p-value is found significant, then it should be significant from classical hypothesis testing point of view, and the expression change should be well beyond the range of the standard error. Finally, the impacted genes and their summary statistics (p-values and effect sizes) are used to compute perturbation factors (detailed below) for the NBIA-prioritized genes and pathways. These perturbation factors are formulated to take into account gene interactions and signal propagation. Through permutation, we construct the null distribution of each pathway, and then compute the p-values of pathways as the fractions that are more extreme than the observed perturbation factors. The identified impacted pathways can be considered as the signature of the disease. This signature can be used for other downstream analyses.

### Effect size and standard error (in Module 1)

Since the datasets are obtained from independent studies, it is reasonable to expect that the expression values are scaled differently in each study. Therefore, it is more reasonable to use standardized mean difference (SMD) as metrics to measure effect sizes, instead of raw mean difference. By default, we use *Hedge’s g*^33^ as the metric to measure expression change between two conditions (see Supplemental Section 1). However, this metric can be substituted by any existing metrics designed for the same purpose.

The central tendency of effect sizes for a gene can be estimated either using a fixed-effects model or a random-effects model^21^. The fix-effects model assumes that there is only one true effect size that underlies all of the studies, and the variability among studies is due to sampling error. This assumption, however, is unlikely to be correct when analyzing multiple independent datasets, since it cannot account for batch effects and heterogeneity between studies^34,35^. In contrast, the random-effects model explicitly takes into consideration the batch effects and data heterogeneity. This model decomposes the variability of effect size estimates into two variance components^35,36^. The first component represents batch effects and data heterogeneity across studies, while the second component represents the variability within each study. In other words, this model includes batch effects and data heterogeneity as a covariate in the designated formula. That is the main reason we favor the random-effects model over the fixed-effects model. See Supplementary Section 3.3, Figs. S5–S8, and Table S5 for more discussion regarding batch effects.

Consider one specific gene and denote *y*_1_, *y*_2_, …, *y*_*m*_ as *Hedge’s g* values computed for *m* studies. We can write the random-effects model as *y*_*i*_ = *μ* + *τ*_*i*_ + *ϵ*_*i*_ with *τ*_*i*_ ~ *N*(0, *σ*^2^) and ${\epsilon }_{i} \sim N(0,{\sigma }_{{\epsilon }_{i}}^{2})$. In this formula, *μ* is the central tendency of the effect size, *τ*_*i*_ represents the term by which the effect size in the *i*^*th*^ study differs from the central tendency, and *ϵ*_*i*_ represents within-study variability. The *τ*_*i*_ variables represent batch effects and data heterogeneity among datasets. The overall effect size *μ* of the gene and its standard error *σ* are estimated iteratively, as described in the literature^35,37–39^. The algorithm stops when further iterations do not change the values of *μ* and *σ*. After the REML algorithm stops, we compute the z-score using the formula $z=\frac{\mu }{\sigma }$ and then calculate the left- and right-tailed p-values of observing such z-score. The obtained *μ* and p-values (*ep*_*l*_ and *ep*_*r*_ where *ep* stands for “effect size p-value”) represent the overall expression change of the gene and the reliability of the estimated effect size.

### Classical hypothesis testing and meta-analysis (in Module 2)

In this work, we apply the linear model and empirical Bayesian test provided by limma^32^ to calculate the two-tailed p-values. We then convert these p-values into one-tailed p-values. We note that this step can be substituted by any other hypothesis testing methods. We favor this empirical approach to avoid relying on strong assumption about the distributions of the expression values. For each gene, the one-tailed p-values are independent and uniformly distributed under the null. We next combine the individual p-values of the *m* studies to obtain one left- and one right-tailed p-value for each gene.

### p-value aggregation (in Module 3)

To combine the p-values obtained from each study, we use the addCLT method that is built on the Central Limit Theorem^29^. This method uses the average of p-values as the test statistic; therefore, it is robust against extreme p-values. Denoting the individual p-values to be combined as *P*_1_, *P*_2_, …, *P*_*m*_, and $X=\frac{{\sum }_{i=1}^{m}{P}_{i}}{m}$, the probability density function (pdf) is derived from a linear transformation of the Irwin-Hall distribution^40,41^: $$f(x)=\frac{m}{(m-1)!}{\sum }_{i=0}^{{\lfloor}m\cdot x{\rfloor}}{(-1)}^{i}\left(\begin{array}{c}m\\ i\end{array}\right){(m\cdot x-i)}^{m-1}$$. When *m* is large, the computation of the Irwin-Hall distribution becomes unstable due to underflow/overflow of memory^29^. In this case, we use the Central Limit Theorem^42^ to estimate this distribution. From the Central Limit Theorem, the average of such *m* independently and identically distributed variables follows a normal distribution with mean $\mu =\frac{1}{2}$ and variance ${\sigma }^{2}=\frac{1}{12m}$, i.e. $X \sim {\mathcal{N}}\left(\frac{1}{2},\frac{1}{12m}\right)$ for large values of *m*. The method is named “addCLT” for “additive-Central Limit Theorem”^29^. See Supplemental Section 1 for details.

### Impacted genes (in Module 3)

After performing effect-size-based meta-analysis and classical hypothesis testing, we have the following statistics for a gene with index *i*: (1) the central tendency *μ*_*i*_ of effect sizes, (2) the left- and right-tailed p-values, *ep*_*il*_ and *ep*_*ir*_, obtained from the z-score (${z}_{i}=\frac{{\mu }_{i}}{{\sigma }_{i}}$ where *σ*_*i*_ is the standard error), and (3) the left and right-tailed p-values obtained from Bayesian statistics, *bp*_*il*_ and *bp*_*ir*_. We further combine the two types of p-values as follows:

$$\begin{array}{rcl}{p}_{il} & = & max(e{p}_{il},b{p}_{il})\\ {p}_{ir} & = & max(e{p}_{ir},b{p}_{ir})\end{array}$$

The intuition behind using maxP^27^ to combine the two types of p-values is to reduce the number of potential false positives. We want to make sure that the selected DE genes are significant from the classical hypothesis testing perspective, as well as have the effect size that is outside the range of standard error. After this, we correct the p-values for multiple comparisons using Benjamini-Hochberg’s False Discovery Rate (FDR)^43^. By default, genes with *FDR* ≤ 1% are considered as genes that are significantly impacted under the effects of the disease. We note that to have a p-value of 1%, the absolute z-score must be at least 2. Therefore, with a cutoff of 1% we choose genes that are not only statistically significant using the empirical Bayesian test, but also have the absolute effect size at least twice the standard error (see Supplementary Sections 3.1 and 3.4 and Figs. S3 and S9 for more discussion about the contribution of each type of p-values and their impact on false positive rate). These p-values and the effect sizes calculated above serve as the input of the Impact Analysis to identify impacted signaling pathways.

### Perturbation factors of genes and pathways (in Module 4)

To identify the biological processes that are impacted by the disease, the Impact Analysis (IA) method^44^ combines two types of evidence: (i) the over-representation of significantly impacted genes in a given pathway, and (ii) the perturbation of the pathway, as measured by propagation expression changes through the network. These two aspects are represented by two p-values: *p*_*de*_ and *p*_*pert*_. The first p-value, *p*_*de*_, is calculated using the hypergeometric model — this probability quantified the over-representation of DE genes in a pathway, compared to the rest of the transcriptome. The second term, *p*_*pert*_, is obtained from an empirical hypothesis testing in which we take into account both the identity of DE genes and their known interactions. It is calculated based on the perturbation factor in each pathway. The perturbation factor (PF) of each gene is defined as: $PF(g)=\Delta E(g)+{\sum }_{u\in U{S}_{g}}{\beta }_{ug}\cdot \frac{PF(u)}{{N}_{ds}(u)}$. The first term, *ΔE*(*g*), captures the signed normalized expression change of the gene, i.e. standardized mean difference (SMD). In the context of meta-analysis, we use the central tendency of effect sizes *μ* to represent *ΔE*(*g*). This value is estimated from multiple studies and is expected to be more robust against noise and bias than the SMD obtained from any single study. The second term is the sum of all PFs of upstream genes, normalized by the number of downstream genes. The value of *β*_*ug*_ quantifies the strength of interaction between *u* and *g*. By default, *β*_*ug*_ = 1 for *activation* and *β*_*ug*_ = −1 for *repression*. The total perturbation in the pathway is then computed as: $PF({P}_{i})={\sum }_{g\in {P}_{i}}PF(g)$.

For each pathway *P*_*i*_, we construct the null distribution of *PF*(*P*_*i*_) by permuting both sample and gene labels. The p-value *p*_*pert*_ is calculated by the fraction of the null distribution of *P*_*i*_ that is more extreme than the observed value. The two p-values, *p*_*de*_ and *p*_*pert*_, are then combined using Fisher’s method to obtain one single p-value for the pathway. This combined p-value represents how likely the pathway is impacted under the effects of the condition^44^. See Supplementary Section 3.2 and Fig. S4 for more discussion.

## Results

Here we analyze 1,737 samples from 27 independent datasets related to Alzheimer’s disease, influenza, and AML. We selected these conditions for our analysis due to two main reasons. First, we were able to find multiple datasets/experiments in public repositories for each of the three diseases. Second, for each disease, there is pathway that was created in KEGG^7^ to describe the known biology and mechanisms of the underlying disease. We use these KEGG pathways to validate the methods and refer to them as *target pathways*. We expect that a good analysis method to identify these *target pathways* as significant. Supplemental Table S1 shows the details of each dataset, including the number of samples, platforms, and tissues. For graphical representation of biological processes, we use the KEGG database version 76, which includes 182 signaling pathways.

We compare NBIA with 4 other pathway analysis approaches: Impact Analysis (IA)^44^, GSA^45^, GSEA^9^, and MetaPath^23^. IA is a topology-aware method while GSEA and GSA are enrichment-based methods. Since IA, GSEA, and GSA are not able to perform meta-analysis, we use addCLT^29^ and Fisher’s method^25^ to combine individual p-values. MetaPath, on the other hand, is a stand-alone meta-analysis method, which performs pathway analysis without the need of any external analysis tool. There are three MetaPath methods: (i) MetaPath_G which performs meta-analysis at the gene level, (ii) MetaPath_P which performs meta-analysis at the pathway level, and (iii) MetaPath_I which combines the results obtained from MetaPath_G and MetaPath_P. In summary, we compare NBIA with 9 different integrative approaches: 6 GSEA-, GSA-, and IA-based approaches, plus 3 MetaPath methods. We consistently set the significance threshold at 5% for all approaches. Pathways with FDR-adjusted p-values smaller than the threshold are consider significantly impacted.

The experimental study consists of two parts. In the first part, we use NBIA for each of the diseases to identify the genes that are consistently differentially expressed. The signature genes and their effect sizes are then used to identify the biological processes at the systems level. We show that NBIA outperforms other approaches: GSEA^9^, GSA^45^, and Impact Analysis^44^ and the MetaPath methods^23^. In the second part, we use the pathway signature identified by NBIA as biomarkers to cluster RNA-Seq data obtained from TCGA for 167 AML patients. We show that the discovered subtypes have significantly different survival profiles using 4 different clustering methods. The Cox p-values obtained from the discovered subtypes equal to 2 × 10^−4^, 3 × 10^−4^, 4 × 10^−5^, and 2 × 10^−6^ for consensus clustering, hierarchical clustering, local shrinkage, and cluster ensemble, respectively. We also show that this would not be possible without knowing the NBIA signature.

### Alzheimer’s disease

There is a target pathway in KEGG, *Alzheimer’s disease*, that describes the known mechanisms and biological processes involved in this disease. However, it is well known that the pathways *Parkinson’s disease* and *Huntington’s disease* share many genes and mechanisms with *Alzheimer’s disease*^46–49^. Therefore, we expect that good analysis methods to identify all of the three neurological disorder pathways as statistically significant and rank them on top.

**Figure 2:**
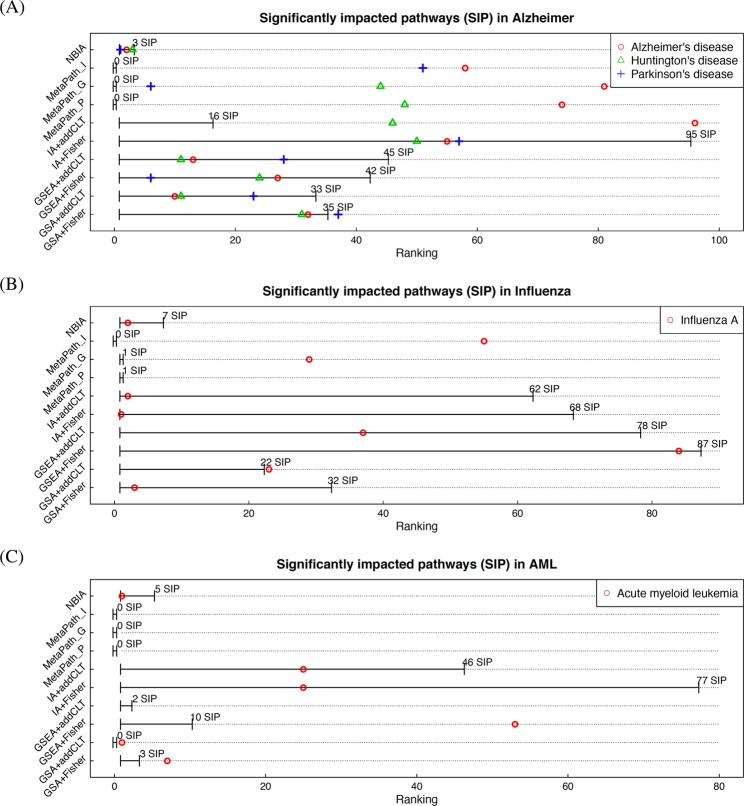
Number of significant pathways and their ranking in Alzheimer’s disease (panel A), influenza (panel B), and AML (panel C) using 10 meta-analysis approaches. The horizontal axis shows the ranking of the pathways while the vertical axis shows the 10 methods. The significance threshold is consistently set to 5% for all approaches. For each method, we draw a segment that represents the range of the significant pathways. For all of the three diseases, MetaPath finds no significant pathway at the significance cutoff of *FDR* = 5%. The 6 GSA-, GSEA-, and IA-based methods generally identify large sets of significant pathways, among which many are likely to be false positives. NBIA consistently identifies the target pathways as significant and ranks them on top in each of the three conditions.

Each of the 10 meta-analysis methods (NBIA, three MetaPath methods, and six GSA-, GSEA-, and IA-based approaches) produces a list of KEGG pathways ranked according to their p-values. Table 1 shows the 10 top ranked pathways and FDR-corrected p-values for NBIA while Supplementary Table S2 shows the 20 top ranked pathways for the other nine methods. Pathways with FDR-corrected p-values less than 5% are considered significant. Figure 2A summarizes the results by showing the number of significant pathways and the ranking of the three neurological disorder pathways for the 10 methods. The horizontal axis shows the ranking of the pathways while the vertical axis shows the 10 methods. For each method, we draw a segment that represents the range of the significant pathways. For example, using NBIA, we identified three significant pathways (Table 1), which are exactly the three neurological disorder pathways. Therefore, the segment for NBIA ranges from 1 to 3 and the three neurological disorders pathways fall onto this segment (top row in Fig. 2A). In another example, using IA + addCLT, we identified 16 pathways as significant (third column in Table S2). Therefore, the segment for IA + addCLT ranges from 1 to 16 in Fig. 2A. The pathway *Alzheimer’s disease* is ranked 96^*th*^ (red circle) and thus falls outside of the segment. Similarly, using GSA + Fisher, we identified 35 significant pathways. The three neurological disorder pathways, *Alzheimer’s disease* (red circle), *Huntington’s disease* (green triangle), and *Parkinson’s disease* (blue plus sign), are ranked at the positions 32^*nd*^, 31^*st*^, and 37^*th*^, respectively. The pathway *Parkinson’s disease* is not significant and thus does not fall onto the segment of significant pathways.

**Table 1:** The top 10 ranked pathways and FDR-corrected p-values obtained by combining Alzheimer’s data using NBIA. The horizontal line represents the cutoff of 5%. All of the three target pathways are ranked on top with FDR-adjusted p-values smaller than 5%.

	NBIA	
	Pathway	p.fdr
1	**Parkinson’s disease**	7e-07
2	**Alzheimer’s disease**	0.0024
3	**Huntington’s disease**	0.0086
4	Glutamatergic synapse	0.1008
5	Amyotrophic lateral sclerosis (ALS)	0.1750
6	Sphingolipid signaling pathway	0.2137
7	Regulation of actin cytoskeleton	0.2137
8	Synaptic vesicle cycle	0.3868
9	Retrograde endocannabinoid signaling	0.6446
10	Fc gamma R-mediated phagocytosis	0.6446

The three MetaPath methods fail to identify the three neurological disorder pathways as the most significant ones. MetaPath_P identifies no significant pathway. The three pathways *Alzheimer’s disease*, *Huntington’s disease*, and *Parkinson’s disease* are ranked at positions 74^*th*^, 48^*th*^, and 121^*st*^, respectively. Similarly, MetaPath_G and MetaPath_I also fail to identify the three neurological disorder pathways as significant. MetaPath_G produces no significant pathway and ranks the three pathways at positions 81^*st*^, 6^*th*^, and 44^*th*^, respectively. In consequences, MetaPath_I also fails to identify the three neurological disorder pathways as significant (adjusted p-values 0.85, 0.87, and 0.85 with rankings 58^*th*^, 83^*rd*^, and 51^*st*^, respectively). IA + addCLT and IA + Fisher, which are topology-aware methods, rank the target pathways very low (not in top 40). IA + addCLT fails to identify any of the three neurological disorder pathways as significant. The GSA-based and GSEA-based methods appear to perform well for this disease. These methods identify the target pathways as significant. However, the two methods also identify a large number of significant pathways, among which many are likely to be false positives.

Finally, we apply NBIA to combine the 10 studies (Table 1). NBIA identifies all of the three neurological disorder pathways as significant and ranks them at the very top. The pathway *Alzheimer’s disease* is ranked 2^*nd*^ with adjusted *p* = 0.002.

#### Influenza

There is a dedicated pathway *Influenza A* that was created in order to describe the known mechanisms involved in the influenza disease. We expect that a good meta-analysis method to identify this target pathway as significant and ranks it among the top impacted pathways. The number of significant pathways and the ranking of the target pathway for the 10 methods are shown in Fig. 2B. Supplemental Table S3 shows the details of top ranked pathways of the competing methods.

MetaPath_P, MetaPath_G and MetaPath_I fail to identify the target pathway as significant and ranks it at the positions 167^*th*^, 29^*th*^ and 55^*th*^, respectively. The two topology-aware methods, IA combined with addCLT and Fisher’s method, identify the pathway *Influenza A* as significant and rank it on top at positions 1^*st*^ and 2^*nd*^, respectively. However, these methods also provide a large set of significant pathways (62 and 68 pathways). Similarly, GSA + Fisher and GSEA + addCLT identify the target pathway as significant but likely to include many false positives as well.

 Table 2 shows the 10 top ranked pathways using NBIA. NBIA finds 7 signifiant pathways with the threshold *FDR* = 5%. The target pathway *Influenza A* is ranked 2^*nd*^ with *FDR* = 8 × 10^−5^. The other significant pathways, *Herpes simplex infection*, *Systemic lupus erythematosus*, *Viral carcinogenesis*, *Pertussis*, *Measles*, and *NOD-like receptor signaling pathway*, are also known to share common mechanisms with influenza and closely associated with immune response of the body^50–53^.

**Table 2:** The top 10 ranked pathways and FDR-corrected p-values obtained by combining influenza data using NBIA. The horizontal line represents the cutoff of 5%. The target pathway *Influenza A* is ranked 2^*nd*^ with an FDR-adjusted p-value of 8 × 10^−5^.

	NBIA	
	Pathway	p.fdr
1	Herpes simplex infection	4e-06
2	**Influenza A**	8e-05
3	Systemic lupus erythematosus	0.0002
4	Viral carcinogenesis	0.0052
5	Pertussis	0.0052
6	Measles	0.0179
7	NOD-like receptor signaling pathway	0.0441
8	Staphylococcus aureus infection	0.0642
9	Cytosolic DNA-sensing pathway	0.0642
10	Alcoholism	0.0642

#### Acute myeloid leukemia

For this disease, the target pathway is *Acute myeloid leukemia*. Again, we use the 10 methods to combine the 8 AML datasets. The ranking and the number of significant pathways are shown in Fig. 2C. The top pathways of the 9 other methods are shown in Supplemental Table S4. Again, the three MetaPath methods identify no significant pathways at the cutoff of 5%. The four GSA- and GSEA-based methods fail to identify the pathway *Acute myeloid leukemia* as significant. IA + addCLT and IA + Fisher succeed in identifying the target pathway as significant but rank it at a relatively low position, 25^*th*^. The 10 top pathways of NBIA are shown in Table 3. The target pathway *Acute myeloid leukemia* is ranked on top with *FDR* = 0.0066.

**Table 3:** The top 10 ranked pathways and FDR-corrected p-values obtained by combining AML data using NBIA. The horizontal line represents the cutoff of 5%. The target pathway *Acute myeloid leukemia* is ranked on top with an FDR-adjusted p-value of 0.0066.

	NBIA	
	Pathway	p.fdr
1	**Acute myeloid leukemia**	0.0066
2	Neurotrophin signaling pathway	0.0178
3	Non-small cell lung cancer	0.0353
4	Renal cell carcinoma	0.0353
5	Transcriptional misregulation in cancer	0.0384
6	ErbB signaling pathway	0.0628
7	Non-alcoholic fatty liver disease (NAFLD)	0.1461
8	Colorectal cancer	0.1913
9	Insulin resistance	0.2792
10	Endometrial cancer	0.2792

### Subtyping AML data

To further validate the signature identified for AML, we downloaded RNA-Seq data for 167 AML patients. The raw TCGA data was sequenced using Illumina GASeq. The processed data and the overall survival information were downloaded from the Broad Institute’s website http://gdac.broadinstitute.org/.

As we reported above, NBIA identified 5 pathways that are significantly impacted in AML. The total number of genes belonging to these pathways are 364. We simply use these genes as selected features in order to refine the partitioning of the 167 AML patients. The comparison between the partitioning with and without feature selection show that the selected pathways and genes play a crucial role in identifying subtypes with significantly different survival.

**Figure 3:**
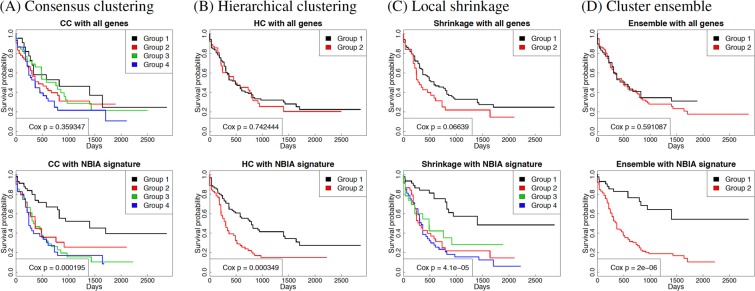
Kaplan-Meier survival analysis of AML subtypes discovered by consensus clustering (A panels), hierarchical clustering (B panels), local shrinkage (C panels), and cluster ensemble (D panels). The top panels show AML subtypes discovered using genome-wide expression values (all genes) while the bottom panels shows the subtypes discovered using genes selected by NBIA. In each panel, the colored curves represent the survival probability of different subtypes. For any of the four methods, we are not able to find subtypes with significantly different profiles when using the genome-wide expression values. In contrast, when applied in conjunction with the pathway signature obtained from NBIA, any of the four methods identifies subtypes with very significant survival profiles. Interestingly, there is one group of patients that are always grouped together in the high-survival group using CC, HC, and local shrinkage. When performing an ensemble of the three partitioning, we are able to separate this group from the rest (panel D). The cluster ensemble algorithm identifies two groups of patients that have very different survival profiles (Cox p-value 2 × 10^−6^). Among the high-survival group, almost 60% of the patients survived at the end of the study (more than 8 years). In contrast, only approximately 10% of the other group survived at the end.

Here we use three existing methods, consensus clustering^54,55^ (CC), hierarchical clustering (HC), and local shrinkage^56^, as well as one newly developed cluster ensemble approach to cluster the gene expression data. We show that using each of the three clustering methods, we discovered subtypes that have significantly different survival profiles. Figure 3 shows the Kaplan-Meier survival analysis^57^ of the discovered subtypes using the four clustering methods. The heatmaps that visualize different subtypes of AML patients on all genes and NBIA signature are shown in Supplementary Fig. S2.

Without feature selection, we are unable to identify subtypes with significant survival differences by using genome-wide expression values. With feature selection, CC is able to find 4 subtypes with Cox p-value = 2 × 10^−4^ while HC finds 2 subtypes with p-value = 3 × 10^−4^. Similarly, the local shrinkage finds 4 subtypes with p-value = 4 × 10^−5^. We note that there is a group of patients that always belongs to the highest-survival group in the three partitionings. The cluster ensemble approach that is designed to look for common pattern between the partitionings is able to separate this group of patients from the rest. This approach identifies two groups of patients with very different survival profiles (Cox p-value = 2 × 10^−6^). Among the high-survival group, almost 60% of the patients survived at the end of the study (more than 8 years). In contrast, only approximately 10% of the other group survived at the end.

We also perform subtyping using the pathway signatures identified by the other meta-analysis methods. The four methods, MetaPath_I, MetaPath_G, MetaPath_P and GSA+addCLT, yield no significant pathway and thus have no pathway signture. The other five methods, IA + addCLT, IA + Fisher, GSEA + addCLT, GSEA + Fisher, and GSA + Fisher, identify 46, 77, 2, 10, and 3 pathways as significant, respectively. We use the pathway signatures of these five methods to subtype AML patients. The Kaplan-Meier survival analysis of the discovered subtypes is shown in Supplementary Fig. S1. The Cox p-values obtained for each analysis are shown in Table 4. Using any of the clustering methods, NBIA has the most significant p-values. In addition, it is the only method that provides significant p-values across all four clustering methods.

**Table 4:** Cox p-values obtained from four clustering methods (consensus clustering, hierarchical clustering, local shrinkage, and cluster ensemble) using seven sets of genes: all genes and the signatures obtained from IA + addCLT, IA + Fisher, GSEA + addCLT, GSEA + Fisher, GSA + Fisher, and NBIA. Cells with emboldening text have p-values smaller than 5%. Using any of the clustering methods, NBIA has the most significant p-values. In addition, it is the only method that provides significant p-values across all four clustering methods.

	All genes	IA + addCLT	IA + Fisher	GSEA + addCLT	GSEA + Fisher	GSA + Fisher	NBIA
Consensus clustering	0.359	**0.016**	**0.001**	0.145	0.089	0.072	**1e-04**
Hierarchical clustering	0.742	**0.004**	**0.002**	**0.009**	0.896	0.345	**3e-04**
Local shrinkage	0.066	**0.002**	0.09	0.788	**0.02**	0.24	**4e-05**
Cluster ensemble	0.591	0.902	**0.048**	0.916	0.068	0.132	**2e-06**

## Conclusion

In this article, we present a novel network-based meta-analysis that is able to combine multiple studies and identify the signaling pathways that are significantly impacted in a given phenotype. The main innovation of NBIA is that it transforms the challenging meta-analysis problem into a set of standard analysis problems that can be solved efficiently. This approach utilizes techniques from both p-value-based and effect-size-based meta-analysis techniques in order to reliably identify a robust set of impacted genes. This set of genes serves as the input of the impact analysis (IA) approach to identify the biological processes that are significantly impacted under the effect of the disease.

To evaluate this framework, we examined 1,737 samples from 27 independent datasets related to Alzheimer’s disease, acute myeloid leukemia (AML), and influenza. NBIA was compared against 9 different approaches, GSA, GSEA, and IA combined with Fisher’s method and addCLT, plus three MetaPath approaches. We demonstrated that NBIA outperforms existing approaches to consistently identify the target pathways as significant and top ranked. We also assessed NBIA’s performance in simulation studies, including Monte Carlo evaluations of batch effects, false positive rates, and discuss the relative contributions of the different quantification steps in the NBIA workflow.

To further validate the framework, we also used the identified signature to cluster RNA-Seq data of 167 AML patients obtained from TCGA. For any of the 4 clustering methods tested, consensus clustering, hierarchical clustering, local shrinkage, and cluster ensemble, the discovered subtypes have significant survival differences with Cox p-value as small as 2 × 10^−6^. Even though our analysis stops at disease subtyping, NBIA can be used for many other applications, such as biomarker detection, drug repurposing, drug synergy, and anti-aging. In each of these areas, identifying the correct set of biological processes that are impacted by the disease/drug is the key for success.

## Supplementary information

Supplementary Materials.
