# Supplementary material for: NBIA: a network-based integrative analysis framework – applied to pathway analysis
Source: Sci Rep. 2020 Mar 6;10:4188. doi: 10.1038/s41598-020-60981-9 (PMC7060280; doi:10.1038/s41598-020-60981-9)
Supplement: Supplementary file 1 — Supplementary Materials. [file 41598_2020_60981_MOESM1_ESM.pdf]

NBIA: a network-based integrative analysis framework – applied to pathway analysis

Supplementary Material

Tin Nguyen[1,*], Adib Shafi[3], Tuan-Minh Nguyen[3], A. Grant Schissler[2], and Sorin Draghici[3]

[1]Department of Computer Science and Engineering, University of Nevada, Reno, NV 89557
[2]Department of Mathematics and Statistics, University of Nevada, Reno, NV 89557
[3]Department of Computer Science, Wayne State University, Detroit, MI 48202

1 Methods

1.1 Fisher's method

Fisher's method [8] is one of the most widely used methods for combining independent p-values. Considering a set of m independent significance tests, the resulting p-values $P_1, P_2, \ldots, P_m$ are independent and uniformly distributed on the interval $[0,1]$ under the null hypothesis. Denoting $X_i = -2\ln P_i$ ($i \in \{1,2,\ldots,m\}$) as new random variables, the cumulative distribution function of X_i can be calculated as follows:

$$\begin{aligned} F_i(x) &= Pr(X_i \leq x) = Pr(-2\ln P_i \leq x) = Pr(P_i \geq e^{\frac{x}{2}}) \\ &= \int_{e^{-\frac{x}{2}}}^{1} f(p)dp = 1 - e^{-\frac{x}{2}} \end{aligned}$$

The above function is the cumulative distribution function of a chi-squared distribution with two degrees of freedom (χ^2_2). Since the sum of chi-squared random variables is also a chi-squared random variable, $-2\sum_{i=1}^{m}\ln(P_i)$ follows a chi-squared distribution with $2m$ degrees of freedom (χ^2_{2m}). In summary, the log product of m independent p-values follows a chi-squared distribution with $2m$ degrees of freedom:

$$X = -2\sum_{i=1}^{m}\ln(P_i) \sim \chi^2_{2m} \tag{1}$$

We note that if one of the individual p-values approaches zero, which is often the case for empirical p-values, then the combined p-value approaches zero as well, regardless of other individual p-values. For example, if $P_1 \to 0$, then $X \to \infty$ and therefore, $Pr(X) \to 0$ regardless of $P_2, P_3, \ldots, P_m$. Therefore, we see that Fisher's method is sensitive to outliers.

1.2 add-CLT

The additive method [9, 11, 7] uses the sum of the p-values as the test statistic, instead of the log product. Let us denote the p-values resulting from the m independent significance tests as $P_1, P_2, \ldots, P_m$. These p-values are independent and uniformly distributed between zero and one under the null (i.e. all p-values between zero and one are equally probable when the null hypothesis is true). Denote the sum of these p-values, $X = \sum_{i=1}^{m} P_i$ ($X \in [0,m]$), as the new random variable. X is known to follow the Irwin-Hall distribution [9, 11] with the following probability density function (pdf):

$$f(x) = \frac{1}{(m-1)!}\sum_{i=0}^{\lfloor x \rfloor}(-1)^i\binom{m}{i}(x-i)^{m-1} \tag{2}$$

Unlike Fisher's method, the additive method is not sensitive to small individual p-values. However, we note that the additive method faces a different practical problem. For large values of m, Equation (2) involves some intensive computation due to a sum of combinatorial and division by a factorial, the result of which can lead to an "arithmetic underflow". Here we describe an enhancement to the additive method that makes it more reliable for larger values of m. First, we change the random variable from the sum of the p-values to the average of the p-values. Second, when m is large, we replace the additive method with the Central Limit Theorem (CLT). The reason for the modification is that the additive method is accurate for small values of m, while the Central Limit Theorem is more accurate for

large values of m. We select $m = 20$ as a conservative cut-off. In other words, we will use the additive method when $m < 20$, and the Central Limit Theorem when $m \geq 20$.

To show the validity of using the Central Limit Theorem for large m, we define a new random variable $Y = \frac{\sum_{i=1}^{m} P_i}{m}$ ($Y \in [0, 1]$), which is the average of p-values. Since $Y = \frac{X}{m}$, we can derive the probability density function (pdf) of Y using a linear transformation of X as follows:

$$g(y) = \frac{m}{(m-1)!} \sum_{i=0}^{\lfloor m \cdot y \rfloor} (-1)^i \binom{m}{i} (m \cdot y - i)^{m-1} \tag{3}$$

The corresponding cumulative distribution function (cdf) can be calculated as:

$$G(y) = \frac{1}{m!} \sum_{i=0}^{\lfloor m \cdot y \rfloor} (-1)^i \binom{m}{i} (m \cdot y - i)^{m} \tag{4}$$

The variable Y is the mean of m independent and identically distributed (i.i.d.) random variables (the p-values from each individual experiment), that follow a uniform distribution with a mean of $\frac{1}{2}$ and a variance of $\frac{1}{12}$. From the Central Limit Theorem [12], the average of such m i.i.d. variables follows a normal distribution with mean $\mu = \frac{1}{2}$ and variance $\sigma^2 = \frac{1}{12m}$, i.e. $Y \sim \mathcal{N}(\frac{1}{2}, \frac{1}{12m})$ for sufficiently large values of m.

1.3 Standardized mean difference

Consider a study composed of two independent groups, and suppose we wish to compare their means for a given gene. Let $\bar{X}_1$ and $\bar{X}_2$ represent the sample means for that gene in the two groups, n_1 and n_2 the number of samples in each group, and S_{pooled} the pooled standard deviation of the two groups. The pooled standard deviation and the standardized mean difference (SMD) can be estimated as:

$$S_{pooled} = \sqrt{\frac{(n_1 - 1)S_1^2 + (n_2 - 1)S_2^2}{n_1 + n_2 - 2}} \tag{5}$$

$$d = \frac{\bar{X}_1 - \bar{X}_2}{S_{pooled}} \tag{6}$$

The estimation of the standardized mean difference described in Equation (6) is often called Cohen's d [4, 3]. The variance of Cohen's d is given as follows:

$$V_d = \frac{n_1 + n_2}{n_1 n_2} + \frac{d^2}{2(n_1 + n_2)} \tag{7}$$

In the above equation, the first term reflects uncertainty in the estimate of the mean difference, and the second term reflects uncertainty in the estimate of S_{pooled}. The standard error of d is the square root of V_d. We note that Cohen's d, which is based on sample averages, tends to overestimate the population effect size for small samples. Let n be the degrees of freedom used to estimate S_{pooled}, i.e. $n = n_1 + n_2 - 2$. The corrected effect size, or Hedges' g [10], can be computed as follows:

$$J = \frac{\Gamma(\frac{n}{2})}{\sqrt{\frac{n}{2}}\Gamma(\frac{n-1}{2})} \tag{8}$$

$$g = J \cdot d \tag{9}$$

where Γ is the gamma function. In this work, we use Hedge' g as the standardized mean difference (SMD) between disease and control groups for each gene/miRNA.

1.4 False Discovery Rate (FDR)

Throughout the manuscript, we use the Benjamini-Hochberg procedure [1] to adjust the p-values for multiple comparisons. This procedure can be executed using the function *p.adjust (p, method="fdr")* (CRAN stats package) where p is the vector of p-values obtained for all pathways. Notably, this method does not involve permutation or bootstrapping. The p-values are first sorted and ranked. Then, each p-value is multiplied by N (the number of pathways) and divided by its assigned rank to give the adjusted p-values.

2 Results

2.1 Experimental data

We use 182 human signaling pathways extracted as graph objects from KEGG. We use expression data related to Alzheimer's disease (10 datasets), influenza (9 datasets), and acute myeloid leukemia (8 datasets), available at `https://www.ncbi.nlm.nih.gov/geo/`. The total number of samples is 1,737. Table S1 shows the summary information for each dataset, including number of samples, platforms, tissues, etc.

For each dataset, we process the raw data using the *threestep* function from the package *affyPLM* [2]. The parameters used for the *threestep* function are: robust multi-array analysis (RMA) background adjustment, quantile normalization, and median polish summarization. If the raw data are not available, we use the data that are already processed and normalized by the data providers.

For subtyping purpose, we also download RNA-Seq data for AML patients. The processed data are available at the Broad Institute's website `http://gdac.broadinstitute.org/`. The total number of patients for this cohort is 167 and the number of genes is 20,100. We also download the vital status and follow-up information from the same website. The survival information is used to plot the Kaplan-Meier survival curves and to calculate the Cox p-values.

Table S1: Description of the 27 gene expression datasets used in the experimental studies. All of the datasets are available at `https://www.ncbi.nlm.nih.gov/geo/`.

	Dataset	Disease	Control	Case	Tissue	Platform
1	GSE1297	Alzheimer's	9	22	Hippocampus	Affymetrix Human U133A
2	GSE4757	Alzheimer's	10	10	Entorhinal cortex	Affymetrix Human U133+ 2.0
3	GSE5281	Alzheimer's	74	87	Entorhinal cortex, medial temporal gyrus, posterior cingulate, superior frontal gyrus, hippocampus, and primary visual cortex	Affymetrix Human U133+ 2.0
4	GSE12685	Alzhiemer's	8	6	Frontal cortex	Affymetrix Human U133A
5	GSE16759	Alzheimer's	4	4	Parietal lobe	Affymetrix Human U133+ 2.0
6	GSE18309	Alzheimer's	3	3	Peripheral blood mononuclear cell	Affymetrix Human U133+ 2.0
7	GSE28146	Alzheimer's	8	22	Hippocampus	Affymetrix Human U133+ 2.0
8	GSE36980	Alzheimer's	47	32	Frontal cortex, temporal cortex, and hippocampus	Affymetrix Human 1.0 ST
9	GSE39420	Alzheimer's	7	14	Brain tissues	Affymetrix Human 1.1 ST
10	GSE48350	Alzheimer's	173	80	Entorhinal cortex, post-central gyrus, hippocampus, and superior frontal gyrus	Affymetrix Human U133+ 2.0
11	GSE42026	Influenza	33	19	Whole blood	Illumina HumanHT-12 3.0
12	GSE40012	Influenza	36	39	Whole blood	Illumina HumanHT-12 3.0
13	GSE29366	Influenza	12	19	Whole blood	Illumina HumanWG-6 3.0
14	GSE17156	Influenza	17	17	Peripheral blood	Affymetrix Human U133A 2.0
15	GSE21802	Influenza	4	36	Blood	Illumina human-6 2.0
16	GSE27131	Influenza	7	7	Blood	Affymetrix Human 1.0
17	GSE71766	Influenza	51	45	Human bronchial epithelial cells	Affymetrix Human U219
18	GSE34205	Influenza	22	28	Peripheral blood mononuclear cells	Affymetrix Human U133+ 2.0
19	GSE82050	Influenza	15	24	Blood	Agilent SurePrint G3 Human 3.0
20	GSE982	AML	6	9	AML cells, monocytes, and neutrophils	Affymetrix Human U133A
21	GSE12662	AML	30	76	CD34+ cells, promyelocytes, neutrophils, and PR9 cell line	Affymetrix Human U133+ 2.0
22	GSE15061	AML	69	202	Bone marrow	Affymetrix Human U133+ 2.0
23	GSE33223	AML	10	20	Peripheral blood mononuclear cell	Affymetrix Human U133+ 2.0
24	GSE35010	AML	16	15	Hematopoietic stem cells and granulocytic monocytic progenitors	Affymetrix Human 1.0
25	GSE37307	AML	19	30	CD34+, hematopoietic, and testis cells	Affymetrix Human U133A
26	GSE63270	AML	42	62	Bone marrow	Affymetrix Human U133+ 2.0
27	GSE68172	AML	5	72	Blood	Affymetrix Human U133+ 2.0

2.2 Pathway analysis

Here we use 10 different integrative approaches to identify the impacted pathways of the 27 datasets: the proposed NBIA, 6 GSA-, GSEA-, and IA-related approaches, and 3 MetaPath methods. For implementation of the NBIA, we used functions from the following R packages: BLMA [16, 15, 14], ROntoTools [25], limma [20], and metafor [24]. NBIA will be available in the package BLMA's next release.

The top pathways of NBIA are shown in Tables 1, 2, and 3 in the main text while those of the other 9 methods are shown in Tables S2, S3, and S4 in this supplemental document.

Table S2: The 20 top ranked pathways and FDR-corrected p-values obtained by combining Alzheimer's data using 9 different approaches: 3 MetaPath methods and 6 GSA-, GSEA-, and IA-related approaches. The horizontal line shows the 5% cutoff. The pathways *Alzheimer's disease*, *Huntington's disease*, and *Parkinson's disease* are highlighted in green. MetaPath_P, MetaPath_G, and MetaPath_I fail to identify the target pathway *Alzheimer's disease* as significant, and rank it at the positions 74th, 81st, and 58th, respectively. The other six methods, GSA+Fisher, GSA+addCLT, GSEA+Fisher, GSEA+addCLT, IA+Fisher, and IA+addCLT, rank the target pathway at the positions 32nd, 10th, 27th, 13nd, 55th, and 96th, respectively.

	MetaPath_P		MetaPath_G		MetaPath_I	
	Pathway	p.fdr	Pathway	p.fdr	Pathway	p.fdr
1	Circadian rhythm	0.2290	Type II diabetes mellitus	0.1510	Long-term depression	0.2992
2	Long-term depression	0.2537	Renin-angiotensin system	0.3365	Type II diabetes mellitus	0.3020
3	Renal cell carcinoma	0.2589	Circadian rhythm	0.4123	Circadian rhythm	0.3210
4	Allograft rejection	0.2658	Thyroid cancer	0.4975	Dorso-ventral axis formation	0.3405
5	VEGF signaling pathway	0.2758	Acute myeloid leukemia	0.5068	Allograft rejection	0.3480
6	Gap junction	0.2777	Parkinson's disease	0.7621	Renal cell carcinoma	0.3751
7	African trypanosomiasis	0.3056	Amoebiasis	0.7627	Acute myeloid leukemia	0.3758
8	Shigellosis	0.3222	RNA transport	0.7639	Gap junction	0.3819
9	NF-kappa B signaling pathway	0.3308	Natural killer cell mediated cytotoxicity	0.7645	VEGF signaling pathway	0.3884
10	Dorso-ventral axis formation	0.3460	Small cell lung cancer	0.7660	African trypanosomiasis	0.4143
11	Type II diabetes mellitus	0.4171	Rheumatoid arthritis	0.7663	Thyroid cancer	0.4287
12	Endocrine and other factor-regulated calcium reabsorption	0.4350	Aldosterone synthesis and secretion	0.7680	Endocrine and other factor-regulated calcium reabsorption	0.4295
13	Long-term potentiation	0.4376	Proteoglycans in cancer	0.7680	Renin-angiotensin system	0.4413
14	Epithelial cell signaling in Helicobacter pylori infection	0.5466	Basal cell carcinoma	0.7681	NF-kappa B signaling pathway	0.4641
15	Glutamatergic synapse	0.5494	p53 signaling pathway	0.7682	Shigellosis	0.4692
16	Glioma	0.5576	Vibrio cholerae infection	0.7713	Bladder cancer	0.5827
17	Glucagon signaling pathway	0.5612	AGE-RAGE signaling pathway in diabetic complications	0.7718	Long-term potentiation	0.5969
18	Acute myeloid leukemia	0.5643	mTOR signaling pathway	0.7741	mTOR signaling pathway	0.6023
19	Antigen processing and presentation	0.5660	Cholinergic synapse	0.7742	Graft-versus-host disease	0.6234
20	Inflammatory mediator regulation of TRP channels	0.5782	Complement and coagulation cascades	0.7743	Epithelial cell signaling in Helicobacter pylori infection	0.7282

	GSA+Fisher		GSA+addCLT		GSEA+Fisher	
	Pathway	p.fdr	Pathway	p.fdr	Pathway	p.fdr
1	Retrograde endocannabinoid signaling	0	Retrograde endocannabinoid signaling	0.0036	Ribosome biogenesis in eukaryotes	0
2	Toxoplasmosis	0	Toxoplasmosis	0.0067	Serotonergic synapse	0
3	Long-term depression	0	GABAergic synapse	0.0067	Glutamatergic synapse	0
4	Gap junction	0	Morphine addiction	0.0067	GnRH signaling pathway	0
5	Amphetamine addiction	0	Long-term depression	0.0067	GABAergic synapse	0
6	Vasopressin-regulated water reabsorption	0	Glutamatergic synapse	0.0072	Oocyte meiosis	0
7	Staphylococcus aureus infection	0	Gap junction	0.0085	Calcium signaling pathway	0
8	Small cell lung cancer	0	Endocrine and other factor-regulated calcium reabsorption	0.0117	Amphetamine addiction	0
9	cAMP signaling pathway	0	Oxytocin signaling pathway	0.0117	VEGF signaling pathway	0
10	Pathogenic Escherichia coli infection	0	Alzheimer's disease	0.0117	Aldosterone-regulated sodium reabsorption	0
11	Platelet activation	0	Huntington's disease	0.0117	Choline metabolism in cancer	0
12	Phospholipase D signaling pathway	0	Synaptic vesicle cycle	0.0117	Dopaminergic synapse	0
13	Adipocytokine signaling pathway	0	Dopaminergic synapse	0.0124	Amyotrophic lateral sclerosis (ALS)	0
14	Ovarian steroidogenesis	0	Circadian entrainment	0.0124	Parkinson's disease	0
15	Maturity onset diabetes of the young	0	Cardiac muscle contraction	0.0129	Sphingolipid signaling pathway	0
16	mRNA surveillance pathway	0	Inflammatory bowel disease (IBD)	0.0140	Cytokine-cytokine receptor interaction	0
17	Chemokine signaling pathway	0	Epithelial cell signaling in Helicobacter pylori infection	0.0140	Carbohydrate digestion and absorption	0
18	Glutamatergic synapse	0.0001	Vibrio cholerae infection	0.0174	Taste transduction	0
19	Synaptic vesicle cycle	0.0002	Allograft rejection	0.0179	Osteoclast differentiation	0
20	Dopaminergic synapse	0.0003	Serotonergic synapse	0.0179	Autoimmune thyroid disease	0

	GSEA+addCLT		IA+Fisher		IA+addCLT	
	Pathway	p.fdr	Pathway	p.fdr	Pathway	p.fdr
1	Ribosome biogenesis in eukaryotes	6e-05	Synaptic vesicle cycle	4e-24	Phagosome	4e-05
2	Serotonergic synapse	0.0002	GABAergic synapse	1e-20	Rheumatoid arthritis	0.0001
3	Glutamatergic synapse	0.0002	Retrograde endocannabinoid signaling	1e-18	Proteoglycans in cancer	0.0001
4	Adrenergic signaling in cardiomyocytes	0.0002	Glutamatergic synapse	5e-17	Ras signaling pathway	0.0033
5	GnRH signaling pathway	0.0003	Phagosome	4e-14	Synaptic vesicle cycle	0.0033
6	GABAergic synapse	0.0007	Gastric acid secretion	2e-13	cGMP-PKG signaling pathway	0.0096
7	Circadian entrainment	0.0007	Morphine addiction	1e-12	Glutamatergic synapse	0.0148
8	Non-alcoholic fatty liver disease (NAFLD)	0.0014	Cholinergic synapse	3e-12	Circadian entrainment	0.0148
9	Oocyte meiosis	0.0015	Circadian entrainment	2e-11	Retrograde endocannabinoid signaling	0.0148
10	Calcium signaling pathway	0.0015	Amphetamine addiction	2e-10	Oxytocin signaling pathway	0.0148
11	Huntington's disease	0.0017	Dopaminergic synapse	3e-10	MAPK signaling pathway	0.0148
12	Amphetamine addiction	0.0018	Rheumatoid arthritis	7e-10	GABAergic synapse	0.0189
13	Alzheimer's disease	0.0018	Calcium signaling pathway	2e-09	Endocytosis	0.0189
14	VEGF signaling pathway	0.0038	MAPK signaling pathway	3e-09	Adrenergic signaling in cardiomyocytes	0.0212
15	Signaling pathways regulating pluripotency of stem cells	0.0038	Long-term potentiation	5e-09	HIF-1 signaling pathway	0.0212
16	Retrograde endocannabinoid signaling	0.0038	Neuroactive ligand-receptor interaction	9e-09	cAMP signaling pathway	0.0326
17	Cardiac muscle contraction	0.0038	Staphylococcus aureus infection	1e-08	Morphine addiction	0.0526
18	Aldosterone-regulated sodium reabsorption	0.0063	Oxytocin signaling pathway	1e-08	Platelet activation	0.0665
19	Choline metabolism in cancer	0.0063	Serotonergic synapse	1e-08	Axon guidance	0.0720
20	Endocrine and other factor-regulated calcium reabsorption	0.0073	Axon guidance	1e-08	Endocrine and other factor-regulated calcium reabsorption	0.0720

Table S3: The 20 top ranked pathways and FDR-corrected p-values obtained by combining influenza data using 9 different approaches: 3 MetaPath methods and 6 GSA-, GSEA-, and IA-related approaches. The horizontal line shows the 5% cutoff. The target pathway *Influenza A* is highlighted in green. GSA+Fisher, GSEA+addCLT, IA+Fisher, and IA+addCLT identify the target pathway as significant and rank it at the positions 13th, 37rd, 1st, and 2nd, respectively.

	MetaPath_P		MetaPath_G		MetaPath_I	
	Pathway	p.fdr	Pathway	p.fdr	Pathway	p.fdr
1	Pancreatic cancer	0.0465	Intestinal immune network for IgA production	0.0360	Pancreatic cancer	0.0540
2	Staphylococcus aureus infection	0.0603	mTOR signaling pathway	0.0570	Intestinal immune network for IgA production	0.0635
3	Intestinal immune network for IgA production	0.0760	Asthma	0.0630	Staphylococcus aureus infection	0.0636
4	Vibrio cholerae infection	0.0762	Staphylococcus aureus infection	0.2028	mTOR signaling pathway	0.0772
5	T cell receptor signaling pathway	0.0815	Allograft rejection	0.2306	T cell receptor signaling pathway	0.0968
6	Peroxisome	0.0906	Leishmaniasis	0.2468	Vibrio cholerae infection	0.1026
7	Progesterone-mediated oocyte maturation	0.1098	RNA transport	0.2750	Asthma	0.1120
8	NF-kappa B signaling pathway	0.1111	Antigen processing and presentation	0.2856	Peroxisome	0.1161
9	Epstein-Barr virus infection	0.1386	mRNA surveillance pathway	0.4016	NF-kappa B signaling pathway	0.1574
10	Epithelial cell signaling in Helicobacter pylori infection	0.1431	Type I diabetes mellitus	0.4361	Progesterone-mediated oocyte maturation	0.1599
11	Acute myeloid leukemia	0.1444	Graft-versus-host disease	0.5185	Leishmaniasis	0.1670
12	Renal cell carcinoma	0.1469	Rheumatoid arthritis	0.5318	Allograft rejection	0.1792
13	Malaria	0.1603	Choline metabolism in cancer	0.5979	Acute myeloid leukemia	0.1827
14	Chronic myeloid leukemia	0.1696	Autoimmune thyroid disease	0.6075	Epithelial cell signaling in Helicobacter pylori infection	0.1866
15	Type I diabetes mellitus	0.1769	Inflammatory bowel disease (IBD)	0.6255	Epstein-Barr virus infection	0.1899
16	Bladder cancer	0.1999	Systemic lupus erythematosus	0.6425	Renal cell carcinoma	0.1967
17	Inflammatory bowel disease (IBD)	0.2056	Legionellosis	0.6595	Malaria	0.2111
18	Allograft rejection	0.2070	NOD-like receptor signaling pathway	0.6716	RNA transport	0.2164
19	Bacterial invasion of epithelial cells	0.2433	Malaria	0.6820	Chronic myeloid leukemia	0.2168
20	Platelet activation	0.2476	Bladder cancer	0.8927	Antigen processing and presentation	0.2193

	GSA+Fisher		GSA+addCLT		GSEA+Fisher	
	Pathway	p.fdr	Pathway	p.fdr	Pathway	p.fdr
1	Type I diabetes mellitus	0	Type I diabetes mellitus	0.0002	Allograft rejection	0
2	Viral carcinogenesis	0	Viral carcinogenesis	0.0003	Natural killer cell mediated cytotoxicity	0
3	Hepatitis B	0	Autoimmune thyroid disease	0.0009	Sulfur relay system	0
4	Viral myocarditis	0	Intestinal immune network for IgA production	0.0010	Non-small cell lung cancer	0
5	Rheumatoid arthritis	0	Cell adhesion molecules (CAMs)	0.0010	Cholinergic synapse	0
6	RIG-I-like receptor signaling pathway	0	Allograft rejection	0.0016	Asthma	0
7	Antigen processing and presentation	0	Inflammatory bowel disease (IBD)	0.0016	Inflammatory bowel disease (IBD)	0
8	Herpes simplex infection	0	Hepatitis B	0.0025	Circadian rhythm	0
9	Systemic lupus erythematosus	0	Graft-versus-host disease	0.0025	Estrogen signaling pathway	0
10	Asthma	0	Viral myocarditis	0.0033	Ribosome biogenesis in eukaryotes	0
11	Measles	0	Rheumatoid arthritis	0.0036	Wnt signaling pathway	0
12	Cytosolic DNA-sensing pathway	0	Toxoplasmosis	0.0046	Vibrio cholerae infection	0
13	Influenza A	0	Legionellosis	0.0046	Fanconi anemia pathway	0
14	Pertussis	0	RIG-I-like receptor signaling pathway	0.0046	T cell receptor signaling pathway	0
15	Hepatitis C	0	Antigen processing and presentation	0.0061	Signaling pathways regulating pluripotency of stem cells	0
16	Alcoholism	0	Herpes simplex infection	0.0061	Prolactin signaling pathway	0
17	Toll-like receptor signaling pathway	0	NF-kappa B signaling pathway	0.0107	Non-alcoholic fatty liver disease (NAFLD)	0
18	Transcriptional misregulation in cancer	0	Systemic lupus erythematosus	0.0113	Prostate cancer	0
19	SNARE interactions in vesicular transport	0	Asthma	0.0120	Hippo signaling pathway	0
20	PPAR signaling pathway	0	Measles	0.0128	Cell adhesion molecules (CAMs)	0

	GSEA+addCLT		IA+Fisher		IA+addCLT	
	Pathway	p.fdr	Pathway	p.fdr	Pathway	p.fdr
1	Allograft rejection	5e-07	Influenza A	3e-31	Measles	2e-09
2	Natural killer cell mediated cytotoxicity	3e-05	Systemic lupus erythematosus	2e-30	Influenza A	2e-08
3	Sulfur relay system	0.0004	Herpes simplex infection	4e-28	NF-kappa B signaling pathway	4e-08
4	Non-small cell lung cancer	0.0004	Measles	9e-24	Viral carcinogenesis	6e-08
5	Osteoclast differentiation	0.0004	Staphylococcus aureus infection	6e-22	HTLV-I infection	6e-08
6	Cholinergic synapse	0.0004	Antigen processing and presentation	4e-19	Hepatitis B	1e-07
7	Asthma	0.0004	Asthma	2e-18	Antigen processing and presentation	2e-07
8	Inflammatory bowel disease (IBD)	0.0005	Viral carcinogenesis	5e-16	Epstein-Barr virus infection	4e-07
9	Circadian rhythm	0.0008	Leishmaniasis	3e-15	Intestinal immune network for IgA production	2e-06
10	Amphetamine addiction	0.0009	Epstein-Barr virus infection	3e-15	Staphylococcus aureus infection	3e-06
11	Estrogen signaling pathway	0.0009	Inflammatory bowel disease (IBD)	6e-15	Tuberculosis	4e-06
12	Ribosome biogenesis in eukaryotes	0.0009	Cell cycle	2e-14	Rheumatoid arthritis	4e-06
13	Pathways in cancer	0.0009	Toxoplasmosis	2e-14	Phagosome	1e-05
14	Wnt signaling pathway	0.0009	Phagosome	2e-14	Systemic lupus erythematosus	2e-05
15	Vibrio cholerae infection	0.0009	Transcriptional misregulation in cancer	2e-14	Toxoplasmosis	2e-05
16	Fanconi anemia pathway	0.0009	Cytosolic DNA-sensing pathway	1e-13	Allograft rejection	2e-05
17	T cell receptor signaling pathway	0.0009	Legionellosis	1e-13	Protein processing in endoplasmic reticulum	0.0001
18	Signaling pathways regulating pluripotency of stem cells	0.0012	Tuberculosis	8e-13	Viral myocarditis	0.0001
19	Prolactin signaling pathway	0.0014	RIG-I-like receptor signaling pathway	2e-12	Cell cycle	0.0003
20	Dilated cardiomyopathy	0.0016	Graft-versus-host disease	3e-12	Legionellosis	0.0003

Table S4: The 20 top ranked pathways and FDR-corrected p-values obtained by combining AML data using 9 different approaches: 3 MetaPath methods and 6 GSA-, GSEA-, and IA-related approaches. The horizontal line shows the 5% cutoff. The target pathway *Acute myeloid leukemia* is highlighted in green. Among the 9 methods, only IA+Fisher and IA+addCLT identify the target pathway as significant and rank it at the same position 25th.

	MetaPath_P		MetaPath_G		MetaPath_I	
	Pathway	p.fdr	Pathway	p.fdr	Pathway	p.fdr
1	Neuroactive ligand-receptor interaction	0.9960	Huntington's disease	0.9678	Neuroactive ligand-receptor interaction	1.0000
2	Ribosome biogenesis in eukaryotes	1.0000	AMPK signaling pathway	0.9782	Ribosome biogenesis in eukaryotes	1.0000
3	RNA transport	1.0000	TNF signaling pathway	0.9854	RNA transport	1.0000
4	mRNA surveillance pathway	1.0000	Non-alcoholic fatty liver disease (NAFLD)	0.9964	mRNA surveillance pathway	1.0000
5	RNA degradation	1.0000	Prion diseases	0.9978	RNA degradation	1.0000
6	PPAR signaling pathway	1.0000	Ribosome biogenesis in eukaryotes	1.0000	PPAR signaling pathway	1.0000
7	Fanconi anemia pathway	1.0000	RNA transport	1.0000	Fanconi anemia pathway	1.0000
8	MAPK signaling pathway	1.0000	mRNA surveillance pathway	1.0000	MAPK signaling pathway	1.0000
9	ErbB signaling pathway	1.0000	RNA degradation	1.0000	ErbB signaling pathway	1.0000
10	Ras signaling pathway	1.0000	PPAR signaling pathway	1.0000	Ras signaling pathway	1.0000
11	Rap1 signaling pathway	1.0000	Fanconi anemia pathway	1.0000	Rap1 signaling pathway	1.0000
12	Calcium signaling pathway	1.0000	MAPK signaling pathway	1.0000	Calcium signaling pathway	1.0000
13	cGMP-PKG signaling pathway	1.0000	ErbB signaling pathway	1.0000	cGMP-PKG signaling pathway	1.0000
14	cAMP signaling pathway	1.0000	Ras signaling pathway	1.0000	cAMP signaling pathway	1.0000
15	Cytokine-cytokine receptor interaction	1.0000	Rap1 signaling pathway	1.0000	Cytokine-cytokine receptor interaction	1.0000
16	Chemokine signaling pathway	1.0000	Calcium signaling pathway	1.0000	Chemokine signaling pathway	1.0000
17	NF-kappa B signaling pathway	1.0000	cGMP-PKG signaling pathway	1.0000	NF-kappa B signaling pathway	1.0000
18	HIF-1 signaling pathway	1.0000	cAMP signaling pathway	1.0000	HIF-1 signaling pathway	1.0000
19	FoxO signaling pathway	1.0000	Cytokine-cytokine receptor interaction	1.0000	FoxO signaling pathway	1.0000
20	Sphingolipid signaling pathway	1.0000	Chemokine signaling pathway	1.0000	Sphingolipid signaling pathway	1.0000

	GSA+Fisher		GSA+addCLT		GSEA+Fisher	
	Pathway	p.fdr	Pathway	p.fdr	Pathway	p.fdr
1	Epithelial cell signaling in Helicobacter pylori infection	0	Acute myeloid leukemia	0.3120	Legionellosis	0
2	Salmonella infection	0	NF-kappa B signaling pathway	0.3120	Natural killer cell mediated cytotoxicity	0
3	Fanconi anemia pathway	0	Estrogen signaling pathway	0.3120	Ras signaling pathway	0
4	Transcriptional misregulation in cancer	0.0917	Fc gamma R-mediated phagocytosis	0.3120	Taste transduction	0
5	Leukocyte transendothelial migration	0.1063	Transcriptional misregulation in cancer	0.3120	Huntington's disease	0
6	Fc gamma R-mediated phagocytosis	0.3780	Epithelial cell signaling in Helicobacter pylori infection	0.3182	Epithelial cell signaling in Helicobacter pylori infection	0.0076
7	Acute myeloid leukemia	0.3780	VEGF signaling pathway	0.4481	Endocytosis	0.0079
8	Apoptosis	0.3780	TNF signaling pathway	0.5166	Rap1 signaling pathway	0.0089
9	Chagas disease (American trypanosomiasis)	0.3780	T cell receptor signaling pathway	0.5166	Focal adhesion	0.0204
10	Oxytocin signaling pathway	0.3780	Carbohydrate digestion and absorption	0.5166	Chagas disease (American trypanosomiasis)	0.0454
11	Osteoclast differentiation	0.3780	Bacterial invasion of epithelial cells	0.5166	NOD-like receptor signaling pathway	0.0502
12	VEGF signaling pathway	0.3780	Endocytosis	0.5166	Epstein-Barr virus infection	0.0660
13	NF-kappa B signaling pathway	0.3780	Non-small cell lung cancer	0.5166	FoxO signaling pathway	0.0660
14	TNF signaling pathway	0.3780	Osteoclast differentiation	0.5271	Pathways in cancer	0.0718
15	Cell cycle	0.3795	AMPK signaling pathway	0.6181	Fc epsilon RI signaling pathway	0.0743
16	Endocytosis	0.3854	Natural killer cell mediated cytotoxicity	0.6181	Synaptic vesicle cycle	0.0847
17	Chemokine signaling pathway	0.5309	MAPK signaling pathway	0.6181	Aldosterone-regulated sodium reabsorption	0.0847
18	Estrogen signaling pathway	0.5309	Oxytocin signaling pathway	0.6181	Aldosterone synthesis and secretion	0.0885
19	p53 signaling pathway	0.5309	Aldosterone-regulated sodium reabsorption	0.6181	mTOR signaling pathway	0.0920
20	T cell receptor signaling pathway	0.5309	Leukocyte transendothelial migration	0.6181	Influenza A	0.1107

	GSEA+addCLT		IA+Fisher		IA+addCLT	
	Pathway	p.fdr	Pathway	p.fdr	Pathway	p.fdr
1	mTOR signaling pathway	0.0351	Transcriptional misregulation in cancer	7e-22	Transcriptional misregulation in cancer	6e-18
2	Aldosterone-regulated sodium reabsorption	0.0434	Phagosome	1e-13	TNF signaling pathway	0.0004
3	Fc epsilon RI signaling pathway	0.0719	Osteoclast differentiation	2e-11	Chemokine signaling pathway	0.0007
4	Choline metabolism in cancer	0.1125	Leishmaniasis	7e-10	Leishmaniasis	0.0007
5	Focal adhesion	0.1125	Chemokine signaling pathway	1e-09	Leukocyte transendothelial migration	0.0013
6	Pathways in cancer	0.1125	Natural killer cell mediated cytotoxicity	1e-09	Salmonella infection	0.0016
7	PPAR signaling pathway	0.1125	Leukocyte transendothelial migration	4e-08	Endocytosis	0.0020
8	cAMP signaling pathway	0.1125	Tuberculosis	2e-07	Pertussis	0.0022
9	Endocytosis	0.1126	Cell cycle	2e-07	Legionellosis	0.0022
10	Epithelial cell signaling in Helicobacter pylori infection	0.1126	Rheumatoid arthritis	2e-07	Viral carcinogenesis	0.0022
11	Rap1 signaling pathway	0.1126	Salmonella infection	6e-07	Amoebiasis	0.0024
12	Type I diabetes mellitus	0.1126	Legionellosis	1e-06	Staphylococcus aureus infection	0.0024
13	Inflammatory bowel disease (IBD)	0.1126	Viral carcinogenesis	3e-06	Pathways in cancer	0.0024
14	Alzheimer's disease	0.1126	Amoebiasis	5e-06	Neuroactive ligand-receptor interaction	0.0034
15	B cell receptor signaling pathway	0.1126	Pertussis	6e-06	Influenza A	0.0035
16	Thyroid hormone signaling pathway	0.1126	Staphylococcus aureus infection	8e-06	Fc gamma R-mediated phagocytosis	0.0040
17	Tight junction	0.1126	Pathogenic Escherichia coli infection	9e-06	Pathogenic Escherichia coli infection	0.0041
18	Regulation of actin cytoskeleton	0.1126	Endocytosis	1e-05	Hepatitis B	0.0043
19	Synaptic vesicle cycle	0.1126	Fc gamma R-mediated phagocytosis	2e-05	Osteoclast differentiation	0.0045
20	Pathogenic Escherichia coli infection	0.1126	Cytokine-cytokine receptor interaction	2e-05	Epstein-Barr virus infection	0.0048

2.3 Subtyping AML data

Using the pathway signatures of the meta-analysis methods, we perform subtyping on 167 AML samples downloaded from the Broad Institute's website `http://gdac.broadinstitute.org/`. The four methods, MetaPath_I, MetaPath_G, MetaPath_P and GSA+addCLT, yield no significant pathway and thus have no pathway signature. We use the pathway signatures of the remaining six methods to subtype AML patients. We also subtype the AML patients using all genes. The Cox p-values obtained for each analysis are shown in Table 4 in the main text. The Kaplan-Meier survival analysis of the discovered subtypes for all genes and NBIA is shown in Figure 3 in the main text. The Kaplan-Meier survival analysis for the remaining methods is shown in Figure S1.

The heatmaps in Figure S2 visualize different subtypes of AML patients derived from either on all genes and NBIA signature. The left panels in Figure S2 show the the heatmaps of subtypes discovered using all genes while the right panels show those using NBIA signature. In these panels, the columns represent the patients and different colors on the top stripe shows different subtypes. To provide the genes that are most meaningful in defining the subtypes, we also performed an analysis of variance (ANOVA) and selected genes that are most significant. The rows in each panel shows the 30 top genes with the most significant p-values.

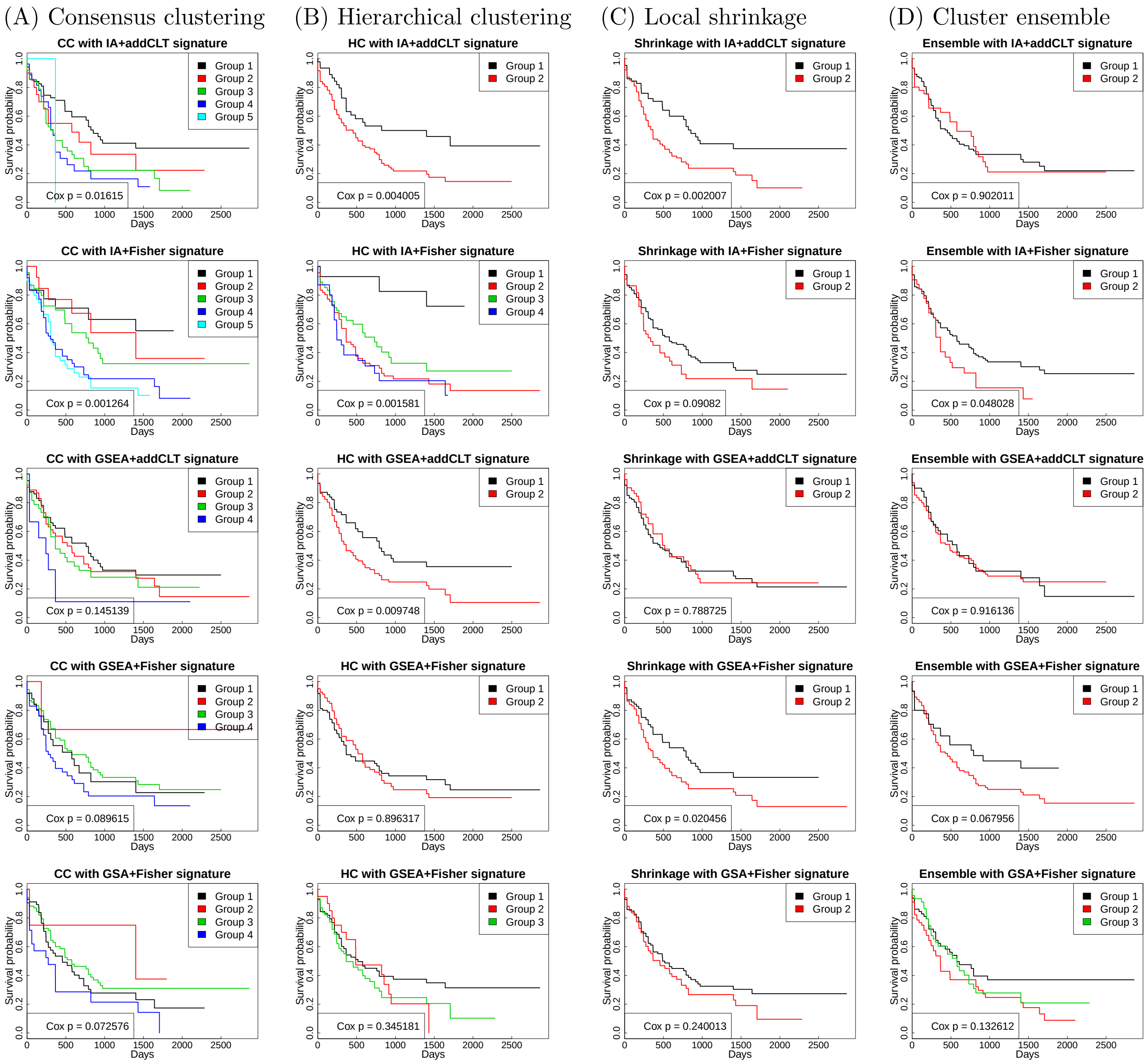

Figure S1: Kaplan-Meier survival analysis of AML subtypes discovered by consensus clustering (A panels), hierarchical clustering (B panels), local shrinkage (C panels), and cluster ensemble (D panels). From top to bottom are the results using the pathway signatures obtained from IA+addCLT, IA+Fisher, GSEA+addCLT, GSEA+Fisher, and GSA+Fisher. In each panel, each colored curve shows the survival probability of each discovered subtype.

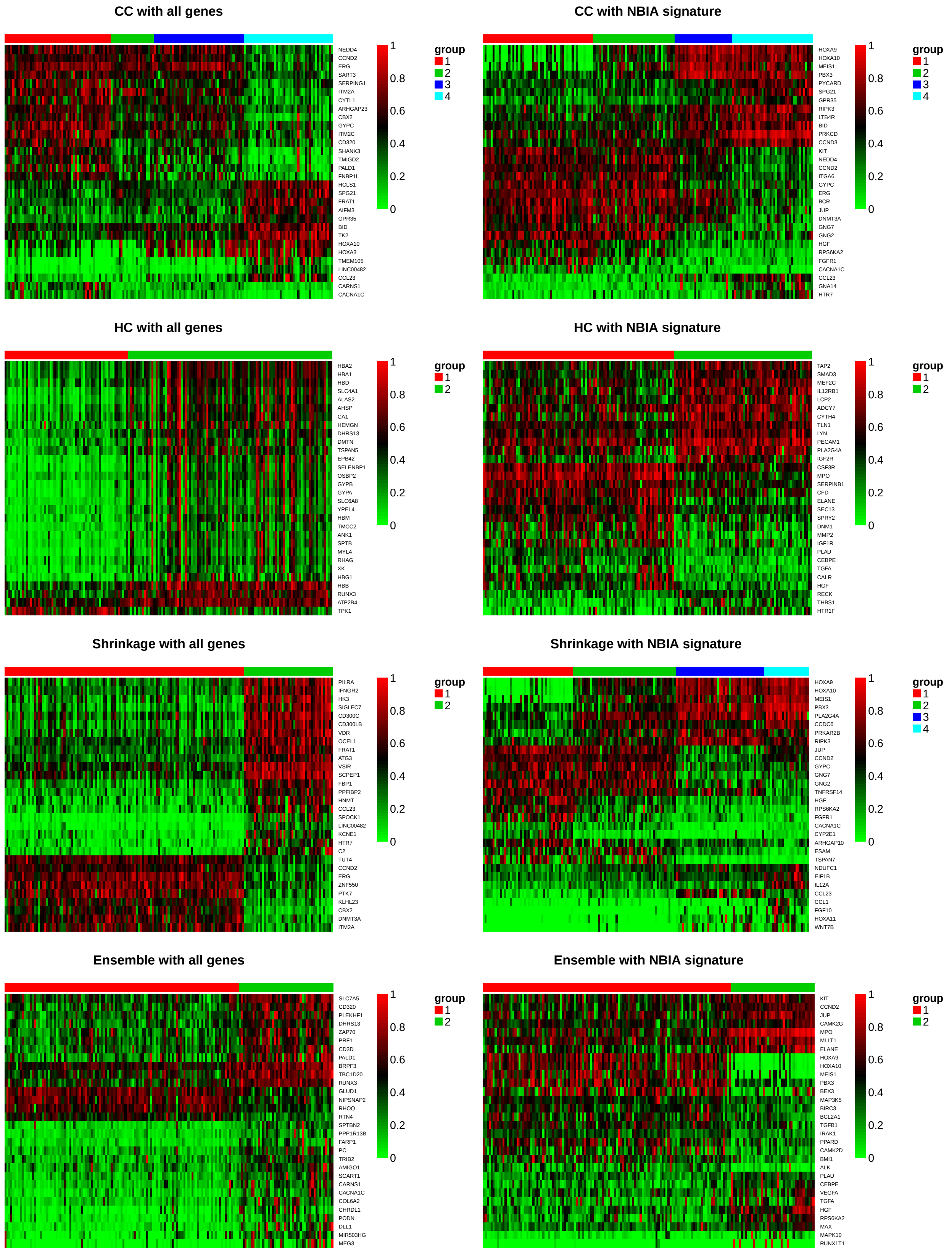

Figure S2: Heatmaps of the subtypes discovered by consensus clustering (CC), hierarchical clustering (HC), local shrinkage, and clustering ensemble. The left panels show the heatmaps of subtypes discovered using genome-wide expression (all genes) while the right panels shows those discovered by using NBIA signature. In each panel, columns represent AML samples while rows represent top 30 genes with the most significant p-values (using ANOVA).

3 Discussions and simulation studies

3.1 On the impact of using both p-addCLT and p-effect size

The intuition of using both p-addCLT and p-effect-size is to combine the two types of p-values in order to reduce potential false positives. We want to make sure that the identified differentially expressed genes are not only significant from the classical hypothesis testing perspective, but also have estimated effect sizes (expression change) that are outside the range of standard errors. By default, genes with both of the p-addCLT and p-effect-size smaller than the threshold of $FDR = 1\%$ are considered as differentially expressed. We note that to have a p-value of 1%, the absolute z-score must be at least 2 ($z = \frac{\mu}{\sigma}$ where μ is the estimated effect size and σ is the standard error). Therefore, with a cutoff of 1% we choose genes that are not only significant using the empirical Bayesian test, but also have the absolute effect size at least twice the standard error. From another perspective, the rationale of using both p-addCLT and p-effect-size is similar to the differential expression analysis using a volcano plot, which combines a measure of statistical significance from a statistical test with the magnitude of the change. The difference here is that instead of focusing on the magnitude, we focus on confirming that the magnitude of the change is well beyond the margin of error. This also allows us to avoid introducing another threshold for effect sizes.

The contribution of each type of p-values depends on the data. For example, p-addCLT contributes more in Alzheimer's data while p-effect-size contributes more in Influenza and AML data. Figure S3 shows the scatter plots of p-addCLT versus p-effect-size. A gene is considered DE if both p-addCLT and p-effect-size are significant, i.e., the gene belongs to the upper right quarter in the plot. In case of Alzheimer's data (Figure S3A), if we removed p-addCLT from the analysis, then we would have obtained a large number of DE genes (genes in upper and lower right quarters), among which many are potentially false positives. Therefore, we would say that p-addCLT contributes more to the analysis in Alzheimer' data. In the case of AML (Figure S3B), most of DE genes are determined by p-effect-size. Removing p-addCLT from this analysis will make a small difference since there are only 5 genes that belong to the lower right panel. Therefore, we would say p-effect-size contributes more in Influenza and AML data analysis (Figure S3B and C).

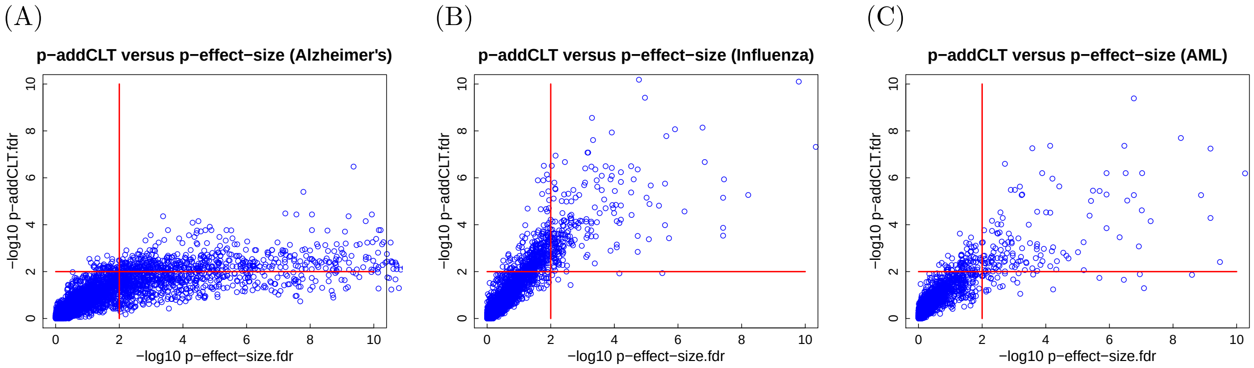

Figure S3: Scatter plot of p-addCLT versus p-effect-size. The horizontal axes represent p-effect-size while the vertical axes represent p-addCLT in the minus log scale. The red lines represent the cutoff of $FDR = 1\%$.

3.2 On the contribution of the hypergeometric model and the perturbation factor model

We expect that each model captures a different type of evidence for differential expression. Therefore, the contribution of each model is expected to be complementary to one another. The hypergeometric model, also known as over-representation analysis [6, 21], estimates the p-value as the probability of obtaining at least the observed number of differentially expressed genes (DE) just by chance. Therefore, it captures the significance of the given pathway from the perspective of the set of genes contained in it. In contrast, the perturbation factor (PF) model aims at capturing the meaningful changes on a given gene topology. This captures the perturbation while taking into consideration the position and role of every gene, and the direction and type of every signal on the pathway. For instance, the insulin processing pathway has the insulin receptor (INSR) as the only entry point in this pathway. Indeed, if the insulin receptor is somewhat disabled, the cell will not be able to process insulin in a normal way and this cell function will be severely disrupted. However, the enrichment analysis will not yield a significant p-value if INSR is the only differentially expressed gene on this pathway. In contrast, the impact analysis will be able to report this

pathway as significantly impacted because it takes into consideration the topology of the pathway and propagates the measured changed of the INSR throughout the rest of the pathway. Thus, the mathematical model employed by the impact analysis will be able to recognize the fact that disabling the entry point in the pathway will shut off the entire pathway [5]. The p-value of the PF model is the probability of obtaining a PF statistic at least as extreme as the one observed under the null hypothesis. A recent very thorough review and benchmarking has shown that the topology based pathway analysis methods are indeed better than the enrichment based methods [17].

We expect the hypergeometric model to play a crucial role in the following scenarios: (i) the gene topology is not available or inaccurate, or (ii) the DE genes are disconnected. In fact, in the those cases, the hypergeometric model is the only meaningful model among the two. However, while useful for the purpose of gene set analysis, this model completely ignores the information about gene topology. In contrast, the PF model aims at fully exploiting all the knowledge about how genes interact as described in the pathway.

Figure S4 illustrates an example analysis of a five-gene pathway. In this example, we monitor a total of 30 genes, among which five are found to be differentially expressed (DE). Two of the DE genes belong to the pathway. Regardless of the position of the DE genes on the pathway, the hypergeometric test provides a p-value of $p_{de} = 0.183$, which is not significant. Now we present two cases in which the positions of the DE genes greatly influence the perturbation factor and its p-value (p_{pert}). In the first case, the DE genes are leaf nodes and cannot perturb the activity of any other genes (the left graph in Figure S4). Gene A does not have any upstream gene nor differentially expressed and therefore $PF(A) = 0$. Similarly, $PF(B) = PF(D) = 0$. For gene C and D, the perturbation factor equals to effect size of the gene, i.e., $PF(C) = PF(D) = 2$. The total perturbation factor (PF) of the pathway is 4. Comparing this total PF against the null distribution constructed for the pathway, we obtain $p_{pert} = 0.272$. Combining p_{de} with p_{pert} using Fisher's method, we obtain a p-value of $p_{comb} = 0.199$, which is not significant.

In the second case, the DE genes have the ability to influence the activity of other genes (the right graph in Figure S4). Again, we start the calculation from gene A. This gene does not have any upstream gene and therefore its perturbation factor is equal to its effect size $PF(A) = 2$. For gene B, $PF(B) = 2 + PF(A) = 4$. For each of the gene C, D, and E, the perturbation factor is one third of gene B. Therefore, $PF(C) = PF(D) = PF(E) = \frac{4}{3}$. The total perturbation factor for the second case is 10. Comparing the total PF against the null, we have $p_{pert} = 0.025$. Combining p_{de} with p_{pert} using Fisher's method, we have $p_{comb} = 0.029$. In summary, the positions of the DE genes play an important role in the PF model. The two obtained p-values greatly differ even when we have the exact same number of DE genes with the same effect size.

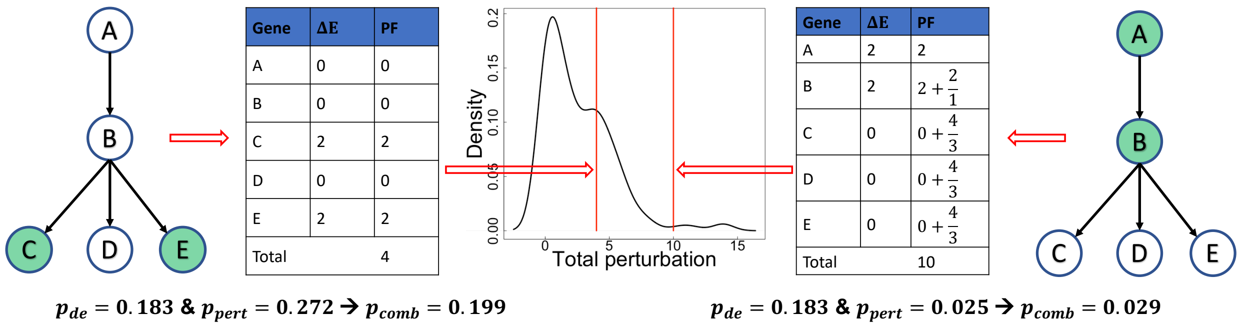

Figure S4: Impact Analysis (IA) using perturbation factor model. The figure shows a five-gene pathway with two differentially expressed (DE) genes in two different situations. In both cases, we have the same number of differentially expressed (DE) genes (marked in green). An ORA would find the two situations equally not significant ($p_{de} = 0.183$ for a set of 30 monitored genes, out of which five are found to be DE). In the first situation (graph in the left), the two DE genes (C and D) are leaf nodes and cannot perturb the activity of any other pathway. In the second situation (graph in the right), the two DE genes (A and B) have the ability to influence the remaining genes in the pathway. This leads to a higher perturbation factor and a more significant p_{pert}.

3.3 On batch effects and data heterogeneity

In the NBIA framework, we estimate the effect sizes of in each dataset/study separately and then combine them using a random-effects model. We use the standardized mean difference as the metric to measure effect size, which standardizes the results of each study to a uniform scale before they can be combined. This metric is designed to be robust against the scale of the original data [10, 4, 3]. In addition, the random-effects model includes batch effects and data heterogeneity in the design: $y_i = \mu + \tau_i + \epsilon_i$. In this formula, μ is the central tendency and τ_i is the term by which the effect size in the ith study different from the central tendency. The τ_i variables represent batch effects and data heterogeneity among datasets [13, 23]. In other words, this model includes batch effects as a

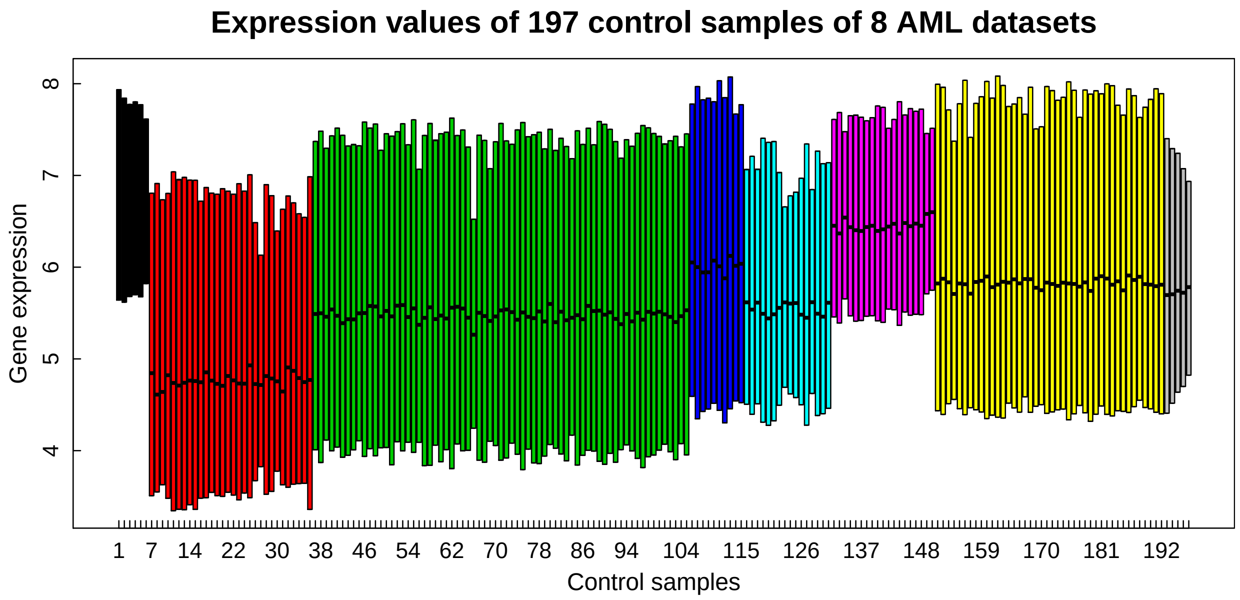

Figure S5: Expression values of 197 control samples obtained from eight AML datasets. The horizontal axis shows the control samples while the vertical axis shows the expression values. Each box represents the range between 25th and 75th percentile of the expression values in each sample. The eight colors represent eight different datasets that the samples originated from. Samples from different datasets have distinctively different expression distributions.

covariate in the designated formula thus explicitly removing the batch effects. That was the main reason we favored the random-effects model over the fixed-effects model when designing NBIA.

To demonstrate that the method is robust against batch effects and data heterogeneity, we simulated a scenario in which data samples were obtained from different data distributions that represent different batches. We first put together 197 control samples obtained from the eight AML datasets analyzed in our manuscript. The data distributions of the 197 samples are shown in Figure S5. As shown in the figure, samples from different datasets have distinctively different expression distributions. These differences represent batch effects and data heterogeneity, including differences between population subgroups (e.g., different ethnicities, gender, race, or living conditions). Next, we randomly selected 20 samples from the sample pool and split them into two equal groups: disease and control. We repeated this process ten times to generate ten datasets. Since samples of both "disease" and "control" groups are randomly drawn from the same pool, a good statistical method should see no difference between these random groups.

In the next step, we altered the gene expression of nine specific genes in the *FoxO signaling pathway* for the samples assigned to the "disease" group. Figure S6 shows the *FoxO signaling pathway* that we focused on. The reason for choosing this KEGG pathway in this simulation is that it consists of a number of genes that do not appear in any other KEGG pathways, thus avoiding cross-talk effects between pathways [5]. The entries marked in red consist of genes that only appear on this pathway: (1) SET9 (gene symbol SETD7 with Entrez ID 80854), (2) FOXO (FOXO6/100132074), (3) Plk (PLK2/10769 and PLK4/10733), (4) KLF2 (KLF2/10365), (5) RAG-1/2 (RAG1/5896 and RAG2/5897), and (6) atrogin-1 (FBXO25/26260 and FBXO32/114907). The actual interactions between these genes are shown in Figure S7, in which SETD7 represses FOX06, and FOX06 activates seven remaining genes. We used the package simPATHy [18] to fit the expression of the ten simulated datasets to the subnetwork shown in Figure S7 and to simulate differential expression for disease samples. In each dataset, the gene SETD7 is down-regulated, leading to the up-regulation of the remaining genes in the subnetwork. The software did not alter expression values of any other genes.

Now we use NBIA to perform a meta-analysis of the ten datasets including the tweaked genes on the *FoxO signaling pathway*. For each gene in each dataset, NBIA calculates: i) a p-value using limma, and ii) an effect size. Next, it combines the p-values across the ten datasets using addCLT to obtain a p-addCLT for each gene. The method also combines the effect sizes to obtain an overall effect size and a p-effect-size for each gene. Figure S8A shows the volcano plot of p-addCLT versus effect size. The horizontal axis represents effect size while the vertical axis represents minus log of FDR-corrected p-addCLT. The figure shows that the gene SETD7 is down-regulated with an approximate effect-size of -1.5 while the other eight genes of the subnetwork are up-regulated with effect

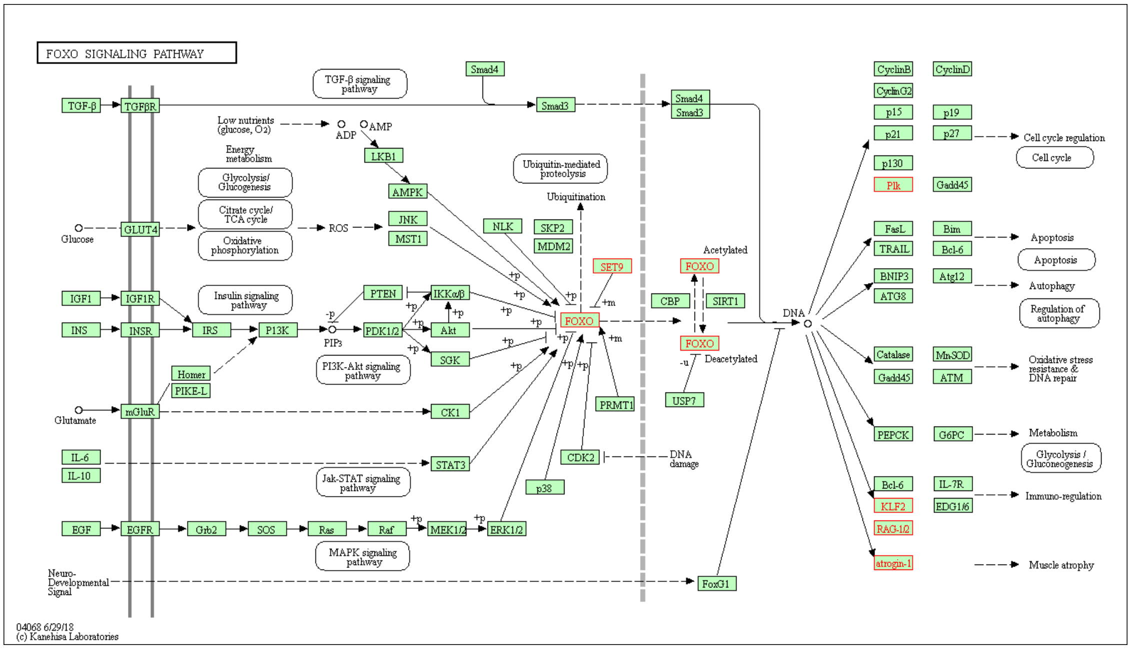

Figure S6: *FoxO signaling pathway*. The entries marked in red consist of genes that do not appear in any other KEGG pathways.

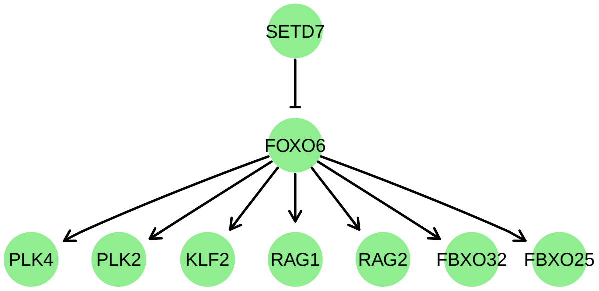

Figure S7: The connected module of *FoxO signaling pathway* that do not appear in any other KEGG pathways.

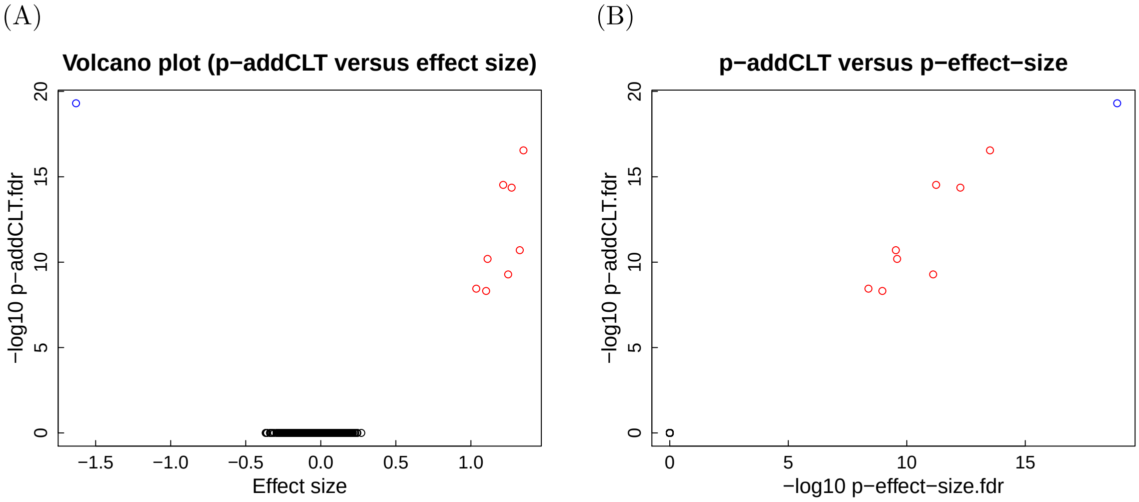

Figure S8: Effect size and p-values obtained for each gene. Panel (A) shows the volcano plot of p-addCLT versus effect size while panel (B) shows the scatter plot of p-addCLT versus p-effect-size. The p-values presented here have been corrected for multiple comparisons using False Discovery Rate (FDR).

Table S5: The 5 top ranked pathways and FDR-corrected p-values obtained from the ten simulated datasets using ten meta-analysis approaches: NBIA, three MetaPath methods, and six GSA-, GSEA-, and IA-related approaches. The horizontal line shows the cutoff of $FDR = 5\%$. The target pathway *FoxO signaling pathway* is highlighted in green. Only NBIA and GSA+Fisher identify the target pathway as significant and rank it on top.

	NBIA		MetaPath_P	
	Pathway	p.fdr	Pathway	p.fdr
1	FoxO signaling pathway	1e-11	Neuroactive ligand-receptor interaction	0.9997
2	Ribosome biogenesis in eukaryotes	1.0000	Ribosome biogenesis in eukaryotes	1.0000
3	RNA transport	1.0000	RNA transport	1.0000
4	mRNA surveillance pathway	1.0000	mRNA surveillance pathway	1.0000
5	RNA degradation	1.0000	RNA degradation	1.0000

	MetaPath_G		MetaPath_I	
	Pathway	p.fdr	Pathway	p.fdr
1	Ovarian steroidogenesis	0.0630	Ovarian steroidogenesis	0.1250
2	Regulation of autophagy	0.1811	Regulation of autophagy	0.3229
3	Regulation of lipolysis in adipocytes	0.1840	Type I diabetes mellitus	0.3454
4	Glutamatergic synapse	0.1960	Staphylococcus aureus infection	0.3470
5	Staphylococcus aureus infection	0.1963	Carbohydrate digestion and absorption	0.3476

	GSA+Fisher		GSA+addCLT	
	Pathway	p.fdr	Pathway	p.fdr
1	FoxO signaling pathway	0	FoxO signaling pathway	0.0668
2	Regulation of actin cytoskeleton	0	p53 signaling pathway	0.3170
3	Fc gamma R-mediated phagocytosis	0	Rap1 signaling pathway	0.3170
4	Tight junction	0	Small cell lung cancer	0.3310
5	Small cell lung cancer	0.2724	Type II diabetes mellitus	0.3839

	GSEA+Fisher		GSEA+addCLT	
	Pathway	p.fdr	Pathway	p.fdr
1	Cocaine addiction	0	Cocaine addiction	0.5811
2	Glutamatergic synapse	0	Protein processing in endoplasmic reticulum	0.9637
3	Maturity onset diabetes of the young	0	SNARE interactions in vesicular transport	0.9637
4	Adipocytokine signaling pathway	0	Non-alcoholic fatty liver disease (NAFLD)	0.9637
5	Protein processing in endoplasmic reticulum	0.5784	Oocyte meiosis	0.9637

	IA+Fisher		IA+addCLT	
	Pathway	p.fdr	Pathway	p.fdr
1	FoxO signaling pathway	1.0000	FoxO signaling pathway	1.0000
2	Ribosome biogenesis in eukaryotes	1.0000	Ribosome biogenesis in eukaryotes	1.0000
3	RNA transport	1.0000	RNA transport	1.0000
4	mRNA surveillance pathway	1.0000	mRNA surveillance pathway	1.0000
5	RNA degradation	1.0000	RNA degradation	1.0000

size of 1 or higher. Figure S8B shows the scatter plot of p-addCLT versus p-effect-size. The genes in the subnetwork have p-values of 10^{-5} or smaller. The p-values of all other genes equal to 1 after FDR correction (both p-addCLT and p-effect-size). Using a default cutoff of $FDR = 1\%$ on both p-addCLT and p-effect-size, NBIA identified exactly the genes in the impacted subnetwork as significant.

Table S5 shows the 5 top ranked pathways and FDR-corrected p-values at the pathway level analysis. NBIA correctly identifies *FoxO signaling pathway* as the only significantly pathway. We also analyzed the data using the other nine meta-analysis approaches: three MetaPath methods and six GSA-, GSEA-, and IA-related approaches. The three MetaPath methods identify no pathway as significant. GSA+Fisher is able to identify the target pathway as significant and ranks it on top. However, this method also identifies three other pathways as significant, which are clearly false positives. The reason is that the p-value for these pathways is zero in one of the datasets and Fisher's method always provides a combined p-value of zero when an individual p-value is zero. GSA+addCLT produces no false positives and also ranks the target pathway on top. However, it is not powerful enough to identify the target pathway as significant. The next method, GSEA+Fisher, produces four false positives. The remaining three methods identify no pathway as significant.

3.4 Assessing false positive rate via simulation

In order to assess the false positive rate of NBIA, we generated datasets under the null hypothesis and then calculated the number of pathways that are identified as significant using NBIA. Similar to the simulation described above, we created a pool of 197 control samples obtained from the eight AML datasets. To simulate a dataset under the null hypothesis, we randomly selected 20 samples from the pool and split them into two equal groups: disease and control. In the first scenario, we set the number of datasets (m) to 5. In this scenario, we generated five datasets and then analyzed the data using NBIA. Pathways with p-values smaller than the significance threshold ($FDR = 5\%$) are considered false positives. The number of significant pathways divided by the total number of pathways is considered false positive rate (FPR). We repeated this process ten times and calculate the average FPR for $m = 5$. In the next scenarios, we increased m and repeated the process described above to compute the average FPR for different values of m.

The average false positive rates for varying values of m are shown in Figure S9A. Overall, NBIA has 0% FPR. As described in the Methods Section, the gene-level analysis produces two lists of p-values – p-addCLT and p-effect-size. These p-values are adjusted using False Discovery Rate (FDR). A gene is considered DE is both of its adjusted p-values are smaller than the threshold of $FDR = 1\%$. These significant genes then serve as input of the Impact Analysis method. Again, the p-values obtained from the pathway-level analysis are adjusted using FDR. Pathways with p-values smaller than the threshold of $FDR = 5\%$ are considered as significant. The 0% false positive rate are mainly due to the rigorous procedure of selecting differentially expressed (DE) genes and the two levels of FDR correction.

To demonstrate the impact of FDR correction steps, we repeated the analysis with the following modifications to NBIA: (i) we removed the FDR adjustment at both gene and pathway levels, and (ii) we set the significance threshold to 5% for both gene- and pathway-level analysis. In this scenario, the FPR is close to the significance threshold of 5% regardless of the number of datasets to be combined (Figure S9B).

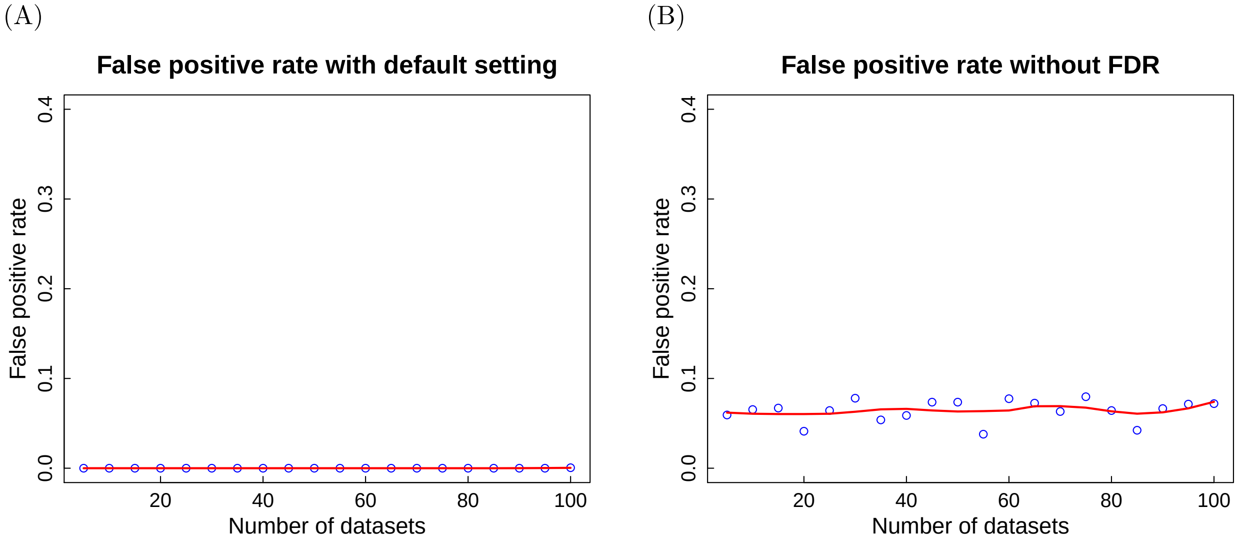

Figure S9: False positive rates of NBIA with varying number of datasets to be combined. (A) False positive rate of NBIA using default settings. (B) False positive rate of NBIA without using False Discovery Rate (FDR) at both gene and pathway levels.

References

[1] Y. Benjamini and Y. Hochberg. Controlling the false discovery rate: A practical and powerful approach to multiple testing. *Journal of The Royal Statistical Society B*, 57(1):289–300, 1995.

[2] B. M. Bolstad. *Low-level analysis of high-density oligonucleotide array data: background, normalization and summarization*. PhD thesis, University of California, 2004.

[3] M. Borenstein, L. V. Hedges, J. P. Higgins, and H. R. Rothstein. *Introduction to Meta-Analysis*. John Wiley & Sons, New York, 2009.

[4] J. Cohen. *Statistical power analysis for the behavioral sciences*. Academic Press, New York, 2nd edition, 2013.

[5] M. Donato, Z. Xu, A. Tomoiaga, J. G. Granneman, R. G. MacKenzie, R. Bao, N. G. Than, P. H. Westfall, R. Romero, and S. Drăghici. Analysis and correction of crosstalk effects in pathway analysis. *Genome Research*, 23(11):1885–1893, 2013.

[6] S. Drăghici, P. Khatri, R. P. Martins, G. C. Ostermeier, and S. A. Krawetz. Global functional profiling of gene expression. *Genomics*, 81(2):98–104, 2003.

[7] E. S. Edgington. An additive method for combining probability values from independent experiments. *The Journal of Psychology*, 80(2):351–363, 1972.

[8] R. A. Fisher. *Statistical methods for research workers*. Oliver & Boyd, Edinburgh, 1925.

[9] P. Hall. The distribution of means for samples of size n drawn from a population in which the variate takes values between 0 and 1, all such values being equally probable. *Biometrika*, 19(3-4):240–244, 1927.

[10] L. V. Hedges and I. Olkin. *Statistical method for meta-analysis*. Academic Press, London, 2014.

[11] J. O. Irwin. On the frequency distribution of the means of samples from a population having any law of frequency with finite moments, with special reference to Pearson's Type II. *Biometrika*, 19(3-4):225–239, 1927.

[12] O. Kallenberg. *Foundations of modern probability*. Springer-Verlag, New York, 2002.

[13] G. A. Milliken and D. E. Johnson. *Analysis of messy data volume 1: designed experiments*, volume 1. Chapman & Hall/CRC, London, 2009.

[14] T. Nguyen, D. Diaz, R. Tagett, and S. Draghici. Overcoming the matched-sample bottleneck: an orthogonal approach to integrate omic data. *Nature Scientific Reports*, 6:29251, 2016.

[15] T. Nguyen, C. Mitrea, R. Tagett, and S. Draghici. DANUBE: Data-driven meta-ANalysis using UnBiased Empirical distributions - applied to biological pathway analysis. *Proceedings of the IEEE*, 105(3):496–515, 2017.

[16] T. Nguyen, R. Tagett, M. Donato, C. Mitrea, and S. Draghici. A novel bi-level meta-analysis approach-applied to biological pathway analysis. *Bioinformatics*, 32(3):409–416, 2016.

[17] T.-M. Nguyen, A. Shafi, T. Nguyen, and S. Draghici. Identifying significantly impacted pathways: a comprehensive review and assessment. *Genome Biology*, 20(1):203, 2019.

[18] E. Salviato, V. Djordjilovic, M. Chiogna, and C. Romualdi. simPATHy: a new method for simulating data from perturbed biological PATHways. *Bioinformatics*, 33(3):456–457, 2016.

[19] K. Shen and G. C. Tseng. Meta-analysis for pathway enrichment analysis when combining multiple genomic studies. *Bioinformatics*, 26(10):1316–1323, 2010.

[20] G. K. Smyth. Limma: linear models for microarray data. In R. Gentleman, V. Carey, S. Dudoit, R. Irizarry, and W. Huber, editors, *Bioinformatics and Computational Biology Solutions Using R and Bioconductor*, pages 397–420. Springer, New York, 2005.

[21] S. Tavazoie, J. D. Hughes, M. J. Campbell, R. J. Cho, and G. M. Church. Systematic determination of genetic network architecture. *Nature Genetics*, 22:281–285, 1999.

[22] L. H. C. Tippett. *The methods of statistics*. Williams & Norgate, London, 1931.

[23] W. Viechtbauer. Bias and efficiency of meta-analytic variance estimators in the random-effects model. *Journal of Educational and Behavioral Statistics*, 30(3):261–293, 2005.

[24] W. Viechtbauer. Conducting meta-analyses in R with the metafor package. *Journal of Statistical Software*, 36(3):1–48, 2010.

[25] C. Voichita and S. Draghici. *ROntoTools: R Onto-Tools suite*, 2013. R package.

[26] B. Wilkinson. A statistical consideration in psychological research. *Psychological Bulletin*, 48(2):156, 1951.
